# Quantitative Proteomics of Human Heart Samples Collected *In Vivo* Reveal the Remodeled Protein Landscape of Dilated Left Atrium Without Atrial Fibrillation

**DOI:** 10.1074/mcp.RA119.001878

**Published:** 2020-04-14

**Authors:** Nora Linscheid, Pi Camilla Poulsen, Ida Dalgaard Pedersen, Emilie Gregers, Jesper Hastrup Svendsen, Morten Salling Olesen, Jesper Velgaard Olsen, Mario Delmar, Alicia Lundby

**Affiliations:** 1 Department of Biomedical Sciences, Faculty of Health and Medical Sciences, University of Copenhagen, Copenhagen N, Denmark; 2 Laboratory for Molecular Cardiology, the Heart Centre, Rigshospitalet, Denmark; 3 The Novo Nordisk Foundation Center for Protein Research, Faculty of Health and Medical Sciences, University of Copenhagen, Copenhagen N, Denmark; 4 Leon H Charney Division of Cardiology, NYU School of Medicine, New York, New York, USA

**Keywords:** Cardiovascular disease, cardiovascular function or biology, clinical proteomics, label-free quantification, tandem mass spectrometry, cardiac proteomics, heart physiology, quantitative proteomics

## Abstract

Genetic and genomic research has greatly advanced our understanding of heart disease. Yet, comprehensive, in-depth, quantitative maps of protein expression in hearts of living humans are still lacking. Using samples obtained during valve replacement surgery in patients with mitral valve prolapse (MVP), we set out to define inter-chamber differences, the intersect of proteomic data with genetic or genomic datasets, and the impact of left atrial dilation on the proteome of patients with no history of atrial fibrillation (AF).

We collected biopsies from right atria (RA), left atria (LA) and left ventricle (LV) of seven male patients with mitral valve regurgitation with dilated LA but no history of AF. Biopsy samples were analyzed by high-resolution mass spectrometry (MS), where peptides were pre-fractionated by reverse phase high-pressure liquid chromatography prior to MS measurement on a Q-Exactive-HF Orbitrap instrument. We identified 7,314 proteins based on 130,728 peptides. Results were confirmed in an independent set of biopsies collected from three additional individuals. Comparative analysis against data from post-mortem samples showed enhanced quantitative power and confidence level in samples collected from living hearts. Our analysis, combined with data from genome wide association studies suggested candidate gene associations to MVP, identified higher abundance in ventricle for proteins associated with cardiomyopathies and revealed the dilated LA proteome, demonstrating differential representation of molecules previously associated with AF, in non-AF hearts.

This is the largest dataset of cardiac protein expression from human samples collected *in vivo*. It provides a comprehensive resource that allows insight into molecular fingerprints of MVP and facilitates novel inferences between genomic data and disease mechanisms. We propose that over-representation of proteins in ventricle is consequent not to redundancy but to functional need, and conclude that changes in abundance of proteins known to associate with AF are not sufficient for arrhythmogenesis.

## Abstract (precis)

Proteomes were measured from human heart biopsies from right atria, left atria and left ventricle of seven male patients with mitral valve regurgitation at a depth of ∼7000 proteins. Results were confirmed in an independent set of biopsies from three individuals. Comparative analysis against data from post-mortem samples showed greatly enhanced quantitative power and confidence level in samples collected from living hearts. Our analysis, combined with data from genome wide association studies, suggest candidate gene associations to mitral valve prolapse.

## Graphical Abstract

**Figure:**
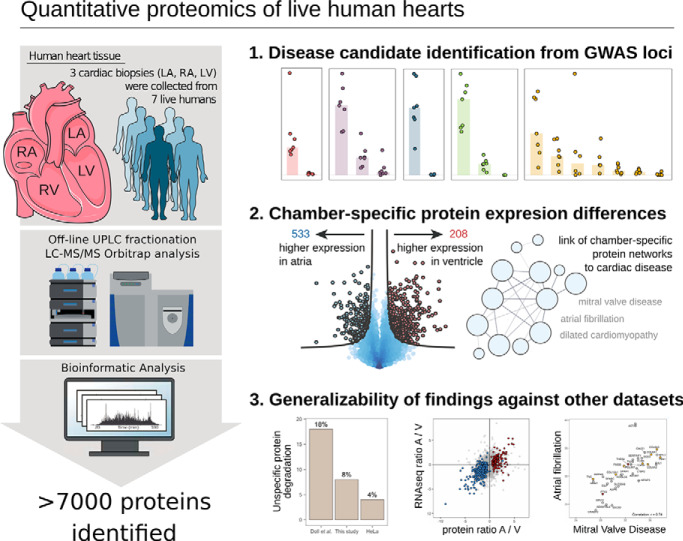

**Highlights**

- Proteomes measured from human heart biopsies collected *in-vivo* covers >7000 cardiac proteins and highlight hundreds of chamber-specific molecular signatures that meaningfully reflect the specialized functions of the respective chambers.
- Protein quantification from freshly collected biopsies is preferential to necropsy samples because of unspecific post-mortem protein degradation in the latter.
- Increased abundances of proteins associated with sustained atrial fibrillation are not a sufficient condition to generate the disease state.
- Protein abundance differences between atria and ventricle primarily originate at the level of gene regulation and reflect a functional need.

For centuries, anatomists and physiologists have recognized that structural and functional differences exist between cardiac chambers and that cardiac diseases can affect specific regions of the heart. Yet, our knowledge of the molecular profile of the different cardiac chambers and how it relates to the causes, manifestations or treatment of disease remains limited. Advances in biochemistry and molecular biology, and more recently the availability of high throughput transcriptomics, have improved our understanding of the molecular composition of the heart (1) and its chambers (2). However, interpretation of transcriptomic data is limited by the fact that transcript abundance is an imperfect proxy for abundance and dynamics of the encoded protein. Quantitative high-resolution proteomics offers an unbiased approach to the identification of chamber-specific protein expression patterns and their relation to cardiac function.

Recent large-scale proteomic studies have focused on mapping the human proteome across all major organs (3, 4), but do not provide the necessary resolution for understanding chamber-specific function and pathophysiology. Prior studies have reported unique protein expression patterns in the human heart (5–7). However, those studies relied mainly on necropsy material collected hours after time of death. We therefore set out to obtain an in-depth quantitative study of the protein expression landscape in heart samples collected from living individuals (*i.e.* collected *in vivo*) and its relation to disease states.

Cardiac tissue acquisition in live humans is limited by obvious ethical considerations. On the other hand, mitral valve replacement consequent to mitral valve prolapse (MVP) is a surgical procedure that permits access to tissue from right (RA) and left atria (LA) and from the left ventricle (LV) without added risk. Furthermore, MVP is not a primary cardiac muscle disease and as such, the state of the atrial and ventricular tissue is only deviated from normal to an extent secondary to the valve dysfunction.

Here, we report data obtained from samples collected during mitral valve replacement surgery in seven males. For all patients, chamber dilation was limited to the LA, allowing a comparison of a dilated (LA) *versus* non-dilated (RA) atrial proteome. None of our patients presented persistent atrial fibrillation (AF), thus giving us the opportunity to investigate the proteome of the dilated LA in a non-AF stage. We confirmed our findings on protein changes in the dilated LA in an independent replication experiment analyzing proteomes of three additional individuals undergoing mitral valve replacement surgery. Moreover, we studied tissue from the LA and RA of a patient with persistent atrial fibrillation to assess the abundance of proteins that were separately found to be differentially expressed in the pre-AF stage. The LV of the ten patients undergoing mitral valve surgery included in this study was electrically and structurally within normal boundaries (no arrhythmias; normal chamber dimensions and normal left ventricular ejection fraction). Taking advantage of the latter, we generated for the first time a comprehensive catalogue and comparative proteome of the LV from living hearts. Finally, given that we collected tissue from living humans, we were able to compare our data to those previously published (7), obtained from material collected several hours post-mortem. This comparison allowed us to define the limits of the use of necropsy material to draw conclusions about the proteome of living hearts.

## MATERIALS AND METHODS

For full description of materials and methods, please see the Supplementary Materials.

### 

#### 

##### Experimental Design and Statistical Rationale

Our study is based on seven biological replicates of biopsy samples from three cardiac chambers (LA, RA, LV). Based on 21 samples fractionated into 12 fractions before MS analysis, a total of 252 MS measurements were performed. No technical replicates were performed. MS measurements of each fraction were performed back-to-back in order to minimize technical variability within each measured fraction, and at the same time distribute technical variability evenly across biological replicates. Our results were validated against an independent cohort of three biological replicates from each cardiac chamber where sample acquisition, laboratory workflow and MS measurements were performed completely independently form the original cohort. The number of biological replicates was chosen based on sample availability from the clinic.

Statistical significance of differential protein expression across chambers was determined by volcano plot analysis based on a permutation-based false-discovery rate (FDR) cutoff (8, 9). This FDR approach employs a combination of Student's *t* test *p* value and fold-change enrichment to determine whether a protein is deemed significant, because both low *p* values and high fold changes are indicative of a biologically important finding.

##### Tissues and Peptide Preparation

Tissue biopsies were collected from LA, RA and LV of patients undergoing mitral valve surgery. Tissue samples were snap-frozen in a container with liquid nitrogen while still in the operating room. All patients gave informed consent to the procedure prior to operation and the procedure conform with the principles outlined in the Declaration of Helsinki. Frozen tissue biopsies were homogenized on a Precellys24 homogenizer (Bertin Technologies, France) in tissue incubation buffer (50 mm Tris-HCl pH 8.5, 5 mm EDTA, 150 mm NaCl, 10 mm KCl, 1% Triton X-100, 5 mm NaF, 5 mm beta-glycerophosphate, 1 mm Na-orthovanadate, containing 1× Roche complete protease inhibitor) with ceramic beads (2.8 and 1.4 mm zirconium oxide beads, Precellys). Homogenates were incubated for 2 h at 4 °C (20rpm), centrifuged (15,000 × *g*, 20 min, 4 °C) and soluble fractions transferred to chilled 1.5 ml tubes. Protein was precipitated and resuspended in Guanidine-HCl buffer (Gnd-HCl; 6MGnd-HCl, 50 mm Tris HCl pH 8.5, 5 mm NaF, 5 mm beta-glycerophosphate, 1 mm Na-orthovanadate, containing 1× Roche complete protease inhibitor). Disulfide bridges were reduced and cysteine moieties alkylated by addition of 5 mm Tris(2-carboxyethyl)phosphine (TCEP) and 10 mm chloroacetamide (CAA) and incubation in the dark at room temperature for 15 min. Up to 1 mg protein was digested in-solution by addition of endoproteinase Lys-C (Trichem ApS, Denmark; 1:100 enzyme/protein ratio) for 1.5 h at 30 °C, 750 rpm in the dark, followed by dilution (1:12 with 50 mm Tris-HCl pH8) and digestion with trypsin overnight (14h) at 37 °C, 750rpm (Life technologies, 1:100 enzyme/protein ratio). Reactions were quenched by trifluoroacetic acid. Soluble fractions were desalted and concentrated on C_18_ SepPak columns (Waters, MA).

##### Offline High pH Fractionation of Peptide Samples

Of each sample, 50–100 μg peptide (in 10 μl injection volume) was fractionated by micro-flow reverse-phase ultrahigh pressure liquid chromatograpy (UPLC) on an Dionex UltiMate 3000 UPLC system (Thermo Scientific) equipped with an ACQUITY UPLC CSH C_18_ Column (130Å, 1.7 μm, 1 mm × 150 mm) at 30 μl/min flow rate, essentially as previously described (10). Outflow was collected in 1-min intervals into 12 concatenated fractions in the autosampler.

##### LC-MS/MS Measurements

Fractionated peptide samples were analyzed by online reversed-phase liquid chromatography coupled to a Q-Exactive HF quadrupole Orbitrap tandem mass spectrometer (LC-MS/MS, Thermo Electron, Bremen, Germany). Peptide samples were brought to concentration of 0.2 μg/μl (diluted in 5% ACN, 0.1% TFA) in 96-well microtiter plates and autosampled (5 μl injection volume) into a nanoflow Easy-nLC system (Proxeon Biosystems, Odense, Denmark). Peptide samples were separated on 15 cm fused-silica emitter columns pulled and packed in-house with reversed-phase ReproSil-Pur C_18_-AQ 1.9 μm resin (Dr. Maisch GmbH, Ammerbuch-Entringen, Germany) in a 1 h multi-step linear gradient (0.1% formic acid constant; 2–25% ACN in 45 min, 25–45% ACN in 8min, 45–80% ACN in 3 min) followed by short column re-equilibration (80–5% ACN in 5 min, 5% ACN for 2 min). Column effluent was directly ionized in a nano-electrospray ionization source operated in positive ionization mode and electrosprayed into the mass spectrometer.

Full-MS spectra (375–1500 *m*/*z*) were acquired after accumulation of 3,000,000 ions in the Orbitrap (maximum fill time of 25 ms) at 120,000 resolution. A data-dependent Top12 method then sequentially isolated the most intense precursor ions (up to 12 per full scan) for higher-energy collisional dissociation (HCD) in an octopole collision cell. MS/MS spectra of fragment ions were recorded at resolution of 30,000 after accumulation of 100,000 ions in the Orbitrap (maximum fill time of 45 ms).

##### Data Analysis

Raw MS data was processed using the MaxQuant software (11) version 1.5.3.30 (Max-Planck Institute of Biochemistry, Department of Proteomics and Signal Transduction, Munich) and proteins identified with the built-in Andromeda search engine. All peptides were used for protein quantification, and label-free quantification (LFQ) was performed in MaxQuant with fast LFQ option enabled. Protein identification results were further processed using the Perseus software suite (8).

To remove minor technical variation between samples, quantile normalization of raw intensities was performed based on the Bioconductor R package LIMMA (12).

## RESULTS

### 

#### 

##### Patient Population

All seven patients were male, middle aged (average 50 years), normal weight (average BMI 23.6), and had mitral valve regurgitation with dilated left atria and a normal left ventricular ejection fraction. Details are provided in supplemental Table S1. Details related to patients in the replication cohort as well as related to a patient with atrial fibrillation are included in the same table. Electrocardiographic recordings from the ten patients undergoing mitral valve surgery detected only normal sinus rhythm and patients did not report palpitations or other symptoms suggestive of atrial fibrillation.

##### Sample Acquisition, Preparation and Analysis

**Fig. 1.**
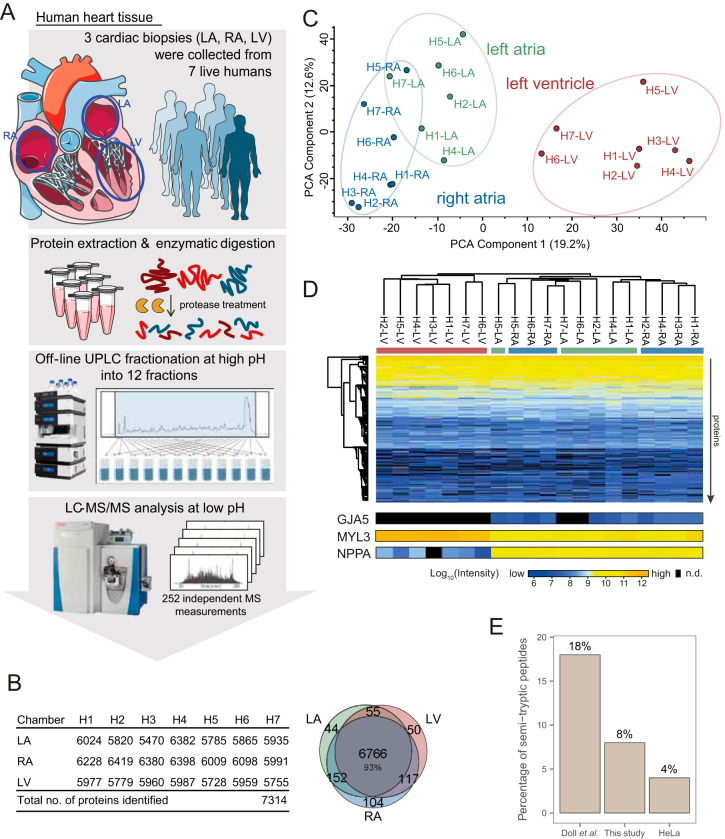
**In-depth human cardiac proteome across chambers identified 7314 proteins from live tissue biopsies.** *A*, High-resolution LC-MS/MS proteomics workflow for deep proteome measurements of freshly isolated cardiac biopsies from seven live individuals undergoing mitral valve surgery. *B*, In total 7,314 proteins were identified across left ventricle, right atrium and left atrium from the seven patients (H1-H7). The Venn diagram shows overlap between protein identifications in each chamber (6766 proteins identified in all chambers). *C*, Principal component analysis (PCA) shows distinction of samples according to chambers, with 19.2% of the variation in the dataset being explained by differences between ventricle and atria along PCA component 1. Separation of right and left atrial samples was observed along components 2 and 4 (12.6%, and 6.8%). *D*, Unsupervised hierarchical clustering of cardiac proteome samples shows clustering of ventricular and atrial samples (columns) according to protein expression profiles (rows). For three proteins with known chamber-specific expression, protein intensities are displayed at the bottom. *E*, Extent of unspecific protein degradation evaluated by fraction of semi-tryptic peptides for cardiac samples collected post mortem (7), our cardiac proteomes, and of a HeLa proteome. *LA*: left atrium, *RA*: right atrium, *LV*: left ventricle, *UPLC*: ultra-high pressure liquid chromatography, *MS*: mass spectrometry, *n.d.*: not detected. Data based on biopsy measurements of seven individuals: 7 RA biopsies, 6 LA biopsies, 7 LV biopsies are underlying this analysis.

Cardiac biopsies were collected from the RA, LA, and LV during mitral valve surgery. The tissue was snap-frozen immediately after collection. Right ventricular samples were not accessible because of the surgical approach. Cardiac samples were homogenized and proteins extracted by detergent-based solubilization followed by enzymatic cleavage by endoproteinase Lys-C and trypsin. The generated peptides were pre-fractionated into 12 fractions (10, 14) followed by high-resolution mass spectrometry measurement of each fraction on a Q-Exactive HF Orbitrap instrument (Fig. 1*A*). Proteome analysis resulted in 130,728 peptides covering 7314 protein groups (Fig. 1*B*). Of these, 6999 proteins were identified by at least 2 peptides (supplemental Table S2). The mass spectrometry (MS) based intensity measurements of protein abundance spanned seven orders of magnitude, highlighting a high dynamic range of the dataset (supplemental Fig. S1*A*). As expected for a comprehensive dataset, the majority of cardiac proteins (6766 or 93%) were identified in all chambers, whereas less than 200 proteins were identified in only one chamber (Fig. 1*B*). Proteins were on average identified based on 23 peptides, 14 of which were unique, resulting in a mean sequence coverage of 37% and mean unique sequence coverage of 30%. The present study provides the largest dataset of cardiac protein expression evaluated from human samples collected *in vivo*.

##### Data Quality and Chamber-specific Data Segregation

To perform a quantitative analysis of protein expression across cardiac chambers, we quantile normalized measured protein intensities (12) and assessed data quality. Our data showed minimal technical variation, as evidenced by only minor differences in intensity distributions prior to normalization and by Pearson correlation coefficients of ≥0.87 for protein intensities across samples (supplemental Fig. S1*A*–S1*B*). Furthermore, most proteins (≥90%) in each cardiac chamber were identified in at least three patient samples, ensuring that comparative analyses across chambers were performed on replicate measurements (supplemental Fig. S1*C*). To evaluate main protein abundance differences between chambers, we performed principal component analysis (PCA). This revealed that biological variation between atria and ventricle was greater than any other difference in the dataset, including disease status, medication or age (*i.e.* 19% of variance in the dataset explained along component 1, Fig. 1*C*). Main drivers of this segregation included well-known marker proteins of the ventricle and the atria (supplemental Fig. S1*D*), such as the atrial natriuretic peptide (NPPA) and Connexin-40 (GJA5) in atrial tissues, and ventricular myosin light chain in the ventricles (MYL3) (Fig. 1*D*). Unsupervised hierarchical clustering of protein intensity profiles across chambers yielded two main clusters separating ventricular and atrial samples, as well as partial separation among right and left atrial samples (Fig. 1*D*). When analyzed independently from the ventricular samples, PCA of left and right atria clustered into two distinct groups (supplemental Fig. S1*E*), thus allowing for differential LA *versus* RA proteome characterization. We assessed the impact of blood protein contamination in the individual chambers and found that blood proteins were present, as expected for tissue samples, but at similar amounts across all chambers (supplemental Fig. S2). Taken together, these results support that our data are of sufficient quality to perform in-depth analyses of global protein expression differences between chambers.

##### Comparative Analysis Against Proteomic Data from Necropsy Samples

A key feature of the cardiac biopsies studied here is that they were collected from living individuals and immediately frozen in the operating room. This contrasts with previous datasets acquired at a similar measurement depth, which were obtained from dead individuals and collected at time of necropsy (7). We found that necropsy material presented increased unspecific proteolysis compared with samples collected *in vivo*. Specifically, analysis of freshly isolated biopsy material leads to less unspecific protein degradation (Fig. 1*E*): 18% of all peptides from necropsy material were semi-tryptic, whereas the same was the case for 8% of the peptides from freshly isolated biopsies. The greater proportion of semi-tryptic peptides in necropsy samples indicate increased unspecific proteolysis because of post-mortem protein degradation. Presence of substantial amounts of degraded peptides, and thus MS precursor peaks, raises concern whether peptides matched on MS1 level through match-between-runs represent degradation products, thereby affecting protein quantifications (supplemental Fig. S3*A*–S3*B*). Quantification of protein abundances based on necropsy biopsies is further challenged by heterogeneous proteolysis across chambers. Overall, our analyses suggest that unspecific protein degradation in necropsy samples can lead to a skewed impact on protein quantification (supplemental Fig. S3*C*–S3*F*) and analysis of proteome data acquired from of post-mortem samples needs to consider unspecific protein degradation. Our analysis suggests that, for studying quantitative differences in protein abundance, analysis of freshly collected tissue biopsies is preferential.

##### Annotation of GWAS Loci Associated with Mitral Valve Prolapse Retrieves Highly Abundant Gene Candidates

**Fig. 2.**
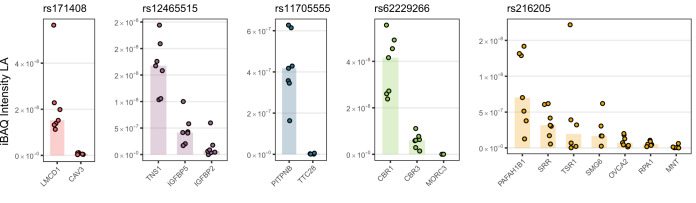
**Protein abundance in patients with mitral valve regurgitation retrieves highly abundant gene candidates in GWAS loci previously associated with mitral valve prolapse.** For genes located in GWAS loci associated with mitral valve prolapse, we retrieved protein intensity measurements from left atrium from patients with mitral valve prolapse (points: single measurements of normalized protein intensities, bars: median). For each locus, high protein abundance singled out 1–2 gene candidates significantly higher expressed in the left atrium, the tissue where the disease manifests. (gene names highlighted in bold, *p* values: two-sided Student's *t* test *p* values). *GWAS*: Genome-wide association study, *MS*: mass spectrometry. Proteomics data based on biopsy measurements of seven individuals: 7 RA biopsies, 6 LA biopsies, 7 LV biopsies are underlying this analysis.

Our data revealed the proteome of the hearts of MVP patients, offering insights into protein expression to complement genomic studies. An existing GWAS data set previously identified six MVP susceptibility loci (Dina *et al.* (15)), yet GWAS cannot directly pinpoint which genes in such genomic loci are causal to a disease. In order to identify which proteins in each of the MVP loci were actually expressed in the heart, we queried our data for their protein expression level in the LA (Fig. 2). Protein abundances across the different proteins were estimated by intensity based absolute quantification (IBAQ) (16). iBAQ is an estimation that allows for abundance comparison across different proteins by correcting for protein size in order to remove MS identification bias of large *versus* small proteins. For four of the loci, we found several orders of magnitude difference between the most abundant and the second-most abundant protein encoded by a gene in the locus. Involvement of a gene in a cardiac phenotype is considered most likely if the gene is transcribed and translated; from that rationale, we suggest that our data offers an alternative way to prioritize genes in GWAS loci. That is, for four of the loci, one protein was considerably more abundant in the LA than any other protein encoded by a gene in the same locus. Thus, for these four loci, our heart proteome data point to a prioritization of the genes LMCD1, TNS1, PITPNB, and CBR1. Notably, functional evidence in support of the involvement of LMCD1 and TNS1 in MVP has been reported (15). For a fifth locus, several proteins were found at similar levels in the left atria, PAFAH1B1, SRR, TSR1, and SMG6. After querying the PheWAS database (17) we consider SMG6 as the most likely of these candidates (supplemental Fig. S4). Taken together, we suggest that genes LMCD1, TNS1, PITPNB, CBR1, and SMG6 are likely candidates underlying the identification of specific loci in the MVP GWAS.

##### Protein Expression Differences Between Atria and Ventricle Reflect Functional Chamber Specialization

**Fig. 3.**
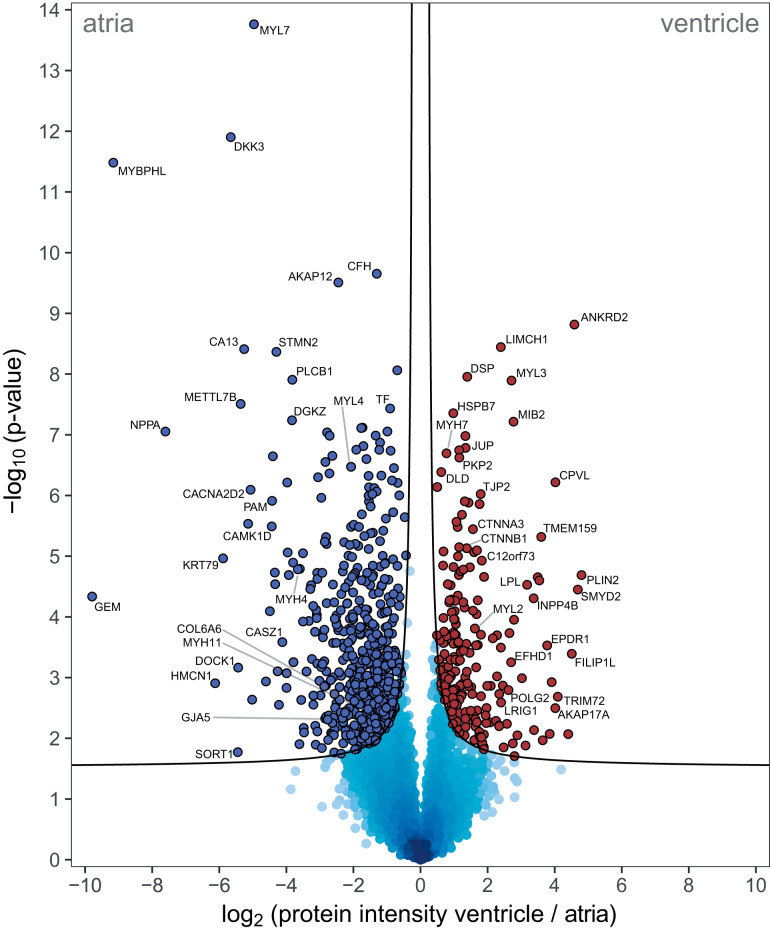
**Differential protein expression between atria and ventricle reflect tissue specialization and reveals its molecular drivers.** Volcano plot analysis of protein expression in atria *versus* ventricle in seven individuals depicts significance of differential expression for each protein (points). Mean fold-change of protein expression between atria and ventricle (left: higher in atria, right: higher in ventricle) is plotted against statistical significance of the differential expression (two-sided Student's *t* test *p* value). Proteins with high differential expression at high statistical significance populate the upper corners of the graph. The analysis identified 741 proteins statistically significantly higher expressed in either atria (533 proteins, blue points) or ventricle (208 proteins, red points) at 5% false discovery rate (black line Data based on biopsy measurements of seven individuals: 7 RA biopsies, 6 LA biopsies, 7 LV biopsies are underlying this analysis.

The greatest biological signal in our dataset was found when comparing the atrial proteome to that of the left ventricle, allowing detailed insights into their functional specialization at the protein level. As will be discussed in a separate section, the vast majority (>98%) of the atrial proteome was not different between RA and LA. We thus analyzed major differences in the atrial *versus* ventricular proteome combining all atrial samples. Global statistical analysis identified a total of 741 proteins significantly over-represented in atria or ventricle (Fig. 3, supplemental Fig. S5, supplemental Table S3). Approximately half of these proteins (366 of them) have previously been reported as differentially expressed ((supplemental Fig. S3*C*), including MYL7 (the atrial isoform of the myosin regulatory chain 2 (18, 19)), DKK3 (expressed in adult atrial myocytes but absent in ventricles (20, 21)), and MYHBPHL (known to have high atrial and low ventricular expression (22)). This consistency with previous findings supported the robustness of our experimental approach and provided additional validation for further studies using the complete dataset.

**Fig. 4.**
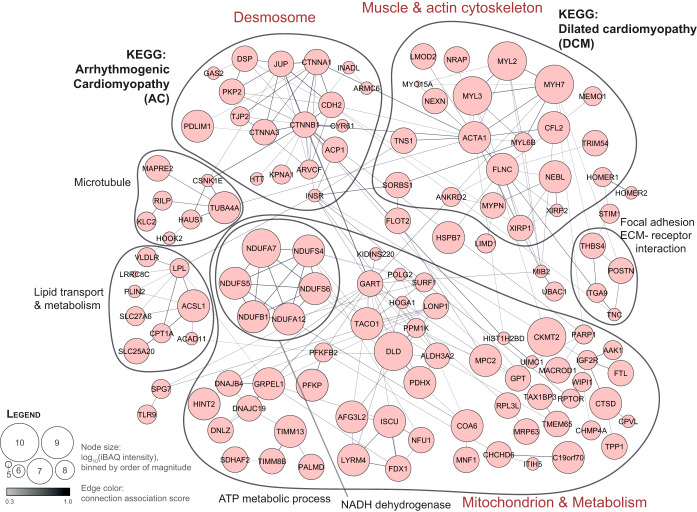
**Protein association network of proteins enriched in human ventricle compared with atria.** Network analysis of proteins significantly enriched in ventricle. A protein-protein interaction network was generated using the STRING database for all proteins with ventricle-specific expression. Proteins are represented by nodes, where the node size reflects measured protein abundance in the ventricle. The network was grouped by clustering of proteins according to strength of the interaction score. For each cluster, gene ontology (GO) terms and KEGG pathway associations were retrieved and resulted in functional enrichments as summarized. KEGG pathway enrichment showed overrepresentation of proteins involved in arrhythmogenic cardiomyopathy (AC) and dilated cardiomyopathy (DCM) in the ventricle. Data based on biopsy measurements of seven individuals: 7 RA biopsies, 6 LA biopsies, 7 LV biopsies are underlying this analysis.

Our results provided a thorough catalogue of proteins differentially expressed in atria or ventricle. We investigated whether these individual differences reflected on differences in chamber-specific protein-protein interacting networks (Fig. 4 and supplemental Fig. S6). We clustered the resulting major protein networks by strength of protein association and performed enrichment analysis on each cluster (23–25). For the atria, enrichment analysis most prominently revealed overrepresentation of muscle (MYH4, MYH11, MYL4, MYL7, MYL10) and actin cytoskeleton (TMOD2, TMOD3, MO1C, ACTR3, CAPN2). Ventricular muscle network analysis also revealed enrichment of muscle and actin cytoskeleton proteins, yet the identity of those proteins was different (MYH7, MYL2, MYL3). Furthermore, the ventricular proteome was enriched for desmosomal/intercellular junction proteins (DSP, PKP2, JUP, TJP2, CTNNA1, CTTNNB1; Fig. 4). And, whereas the atria showed enrichment for metabolic pathways focused on oxidation-reduction processes and small molecule metabolic processes (supplemental Fig. S6), the ventricular network was enriched for mitochondrial and metabolic terms likely indicative of the high-energy demand of the ventricular muscle (Fig. 4).

To evaluate the robustness of our findings we performed an independent validation experiment based on biopsies collected from LA, RA, and LV of an additional set of three individuals undergoing mitral valve surgery. Methods of detection, measurement and analysis were the same as those used in the original series of 7 patients. The results obtained in the replication cohort were highly consistent with those obtained from the original group. Specifically, the correlation coefficients were *r* = [0.91–0.93] for all samples (supplemental Fig. S7*A*–S7*D*). In this dataset we could confirm greater abundance in atria for 477 of the proteins deemed significantly more abundant in atria, and we confirmed greater abundance in ventricles for 178 of the proteins deemed significantly more abundant in the ventricles (supplemental Fig. S7*E*). It is interesting to point out that proteins with preferential expression in the ventricle are also related to diseases that largely affect the ventricular tissue (Fig. 4). For example, mutations in JUP, DSP, PKP2, DSG2, DSC2, CTNNA3, CDH2, or PLN (all of them more abundant in LV) predominantly lead to arrhythmogenic right ventricular cardiomyopathy, and mutations in MYH7, HSPB7, NEXN and MYPN (also all more abundant in LV) lead to dilated cardiomyopathy. In general, proteins encoded by genes with high confidence of genetic DCM causality were found to be more abundant in the left ventricle (supplemental Fig. S8). These observations lead us to propose that higher protein expression in a given chamber does not reflect functional redundancy but rather, it is a manifestation of increased physiological need.

##### Transferability of Proteome Data from Seven Mitral Valve Patients to the General Population

Our data revealed differences in protein expression between the atria and the left ventricle in the population studied. As an indirect test for the robustness of our findings, we compared the experimentally-determined differential proteome with the differential transcriptome (atrium *versus* left ventricle) that can be deduced from the 190 individuals reported in the GTex v7 database (26). As generally observed for transcript and protein abundance of the total population, the correlation between gene expression and protein abundance was weak, with Spearman correlation coefficients of 0.4 for both atria and ventricle (supplemental Fig. S9*A*–S9*B*). Yet, when focusing on the proteins with either ventricle- or atria over-representation in our dataset, we found a preservation of the trend at the transcript level. Specifically, 85% of all atria-specific proteins and 90% of all ventricle-specific proteins showed highest transcript levels in the same chambers in the 190 individuals from GTex (supplemental Fig. S9*C*). Thus, the differences (atria *versus* LV) in protein abundance that we have identified in our MVP patients are likely to be manifest in the general population.

##### The Differential Proteome of the Atria in Patients with Dilated LA but No Evidence of AF

Having validated our dataset, we searched for differences in the proteome between LA and RA in our patient population. It is important to note that our samples were collected from patients with left atrial dilation because of MVP. Thus, although acknowledging that structural and functional differences between normal left and right atria exist (see Discussion), these tissues gave us the unique opportunity to investigate the differential proteome of a dilated atrial chamber (LA) *versus* that of an atrial chamber that was not dilated, namely, the RA.

**Fig. 5.**
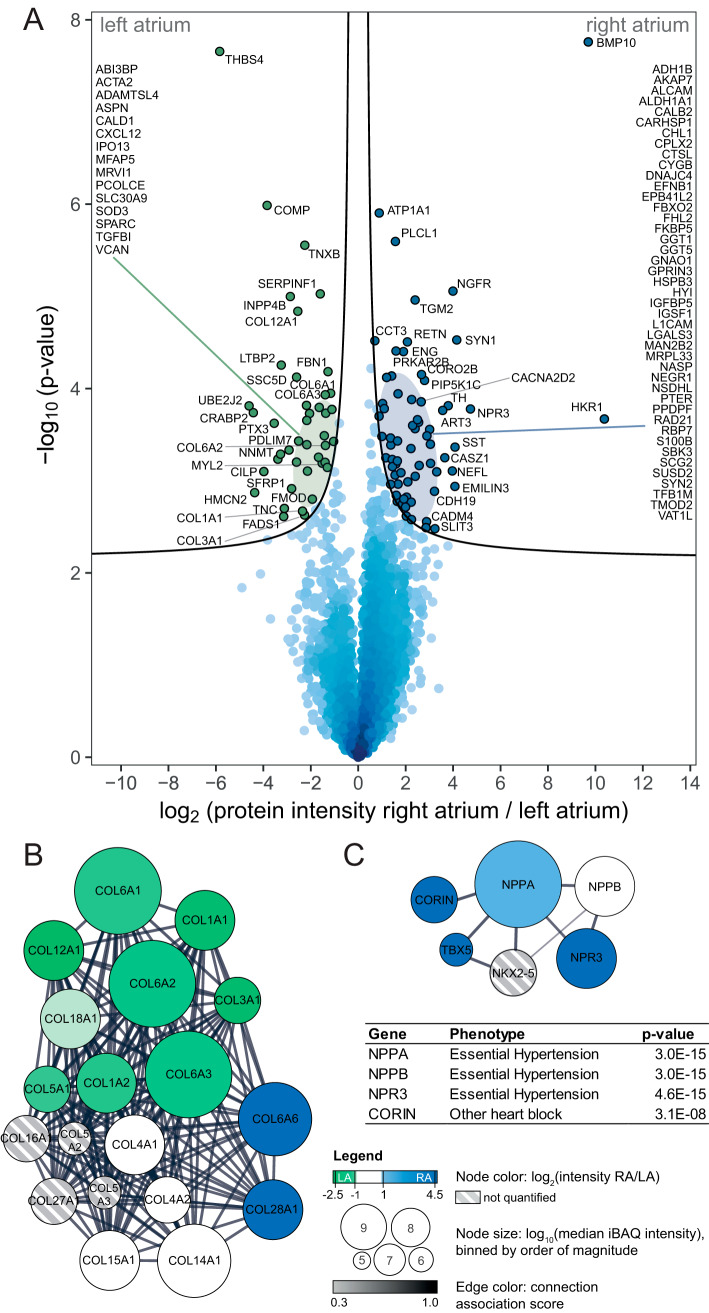
**Differential protein expression between right and left atrium captures physiology and disease status.** *A*, Volcano plot analysis of protein expression in right- *versus* left atria in seven individuals depicts significance of differential expression for each measured protein (points). Mean fold-change of protein expression between RA and LA (left: higher in LA, right: higher in RA) is plotted against statistical significance of the differential expression (Student's *t* test *p* value). Proteins with strong differential expression at high statistical significance populate the upper corners of the graph. The analysis identified 109 proteins statistically significantly higher expressed in either LA (42 proteins, green) or RA (67 proteins, blue) at 5% false discovery rate (black line) FDR. Gene names are listed for all significant proteins. *B* and *C*, STRING-based protein-protein interaction networks for collagens (*B*) and natriuretic peptide system components (*C*) identified in our data depict protein abundance (node size) and differential expression between chambers (node color: green - higher in LA, blue - higher in RA). The networks highlight higher collagen abundance in LA (*B*) and higher abundance of natriuretic peptide signaling pathway in RA (*C*). The table shows PheWAS associations and corresponding *p* values for genes in the natriuretic peptide signaling network from the UK Biobank PheWeb, highlighting involvement in hypertension and heart block. Abbreviations: *LA*: left atrium; *RA*: right atrirum, *iBAQ*: intensity-based absolute quantification. Data based on biopsy measurements of seven individuals: 7 RA biopsies, 6 LA biopsies, 7 LV biopsies are underlying this analysis.

Statistical analysis identified 109 proteins with significantly different expression: 42 over-represented in the LA and 67 in the RA (Fig. 5*A*, supplemental Table S4). From our replication dataset, we confirmed higher expression in LA for 38 of the proteins and we confirmed higher expression in RA for 62 of the proteins (supplemental Fig. S7*F*).

As previously reported, BMP10 was the protein with the most prominent differential expression in favor of the right atria (27). Also as expected, we observed that known protein markers for MVP (28) were highly abundant in left atria (supplemental Fig. S4*A*), the cardiac chamber where the disease manifests. Our dataset was then used to identify other unknown differentials.

The most differentially expressed protein for the dilated left atria was THBS4. In non-diseased states of the heart, transcript levels of THBS4 have been reported predominantly for the ventricle (29), where it is known to regulate fibrosis and remodeling in response to pressure overload (30, 31). Its abundance in the dilated LA indicates that THBS4 may also participate in a pro-fibrotic state consequent to chamber dilation in the LA. We also found differences in the expression of cytokines (such as LTBP2, CXCL12, and TGFBI) and growth factor receptors (such as NGFR) that likely reflect the trajectory of the LA toward a pro-inflammatory, pro-fibrotic state. Gene ontology (GO) enrichment analysis revealed that in the dilated LA there was predominance of molecules involved in extracellular matrix reorganization and fibrosis, including Collagens type I, III, VI, and XII as well as FBN1, FN1, TNC, COMP, VCAN, and FMOD. Furthermore, we found an over-representation of TGFB1, a molecule previously associated not only with fibrosis but also with permanent AF ((32) and see Discussion). Also related to AF -though from the point of view of inflammation - and over-represented in the LA of our patients was pentraxin 3 (PTX3). Finally, we found the abundance of TBX5, a molecule associated with AF and a gene regulatory network that maintains atrial rhythm (33), to be higher in the non-dilated RA.

To evaluate these findings in the context of atrial fibrillation, we collected cardiac tissue from LA and RA from a patient with atrial fibrillation and measured the proteomes from these samples. In supplemental Fig. S10 we show that the abundance of proteins that were found to be significantly more abundant in the dilated LA of non-AF patients are expressed at similar levels in the LA of a patient with AF. As we only have data from one patient with AF, it is not possible to draw general conclusions, but our data suggest that the alterations in the protein expression of the dilated left atrium persists into the disease state of AF and yet, are not sufficient condition to underlie the disease.

##### Other Functional Networks Unveiled by the LA Versus RA Differential Proteome

Our LA *versus* RA dataset revealed other differentially expressed proteins not related to regulation of fibrosis or inflammation. We found enrichment in the RA for proteins involved in neuronal signaling (CHL1, EFNB1, L1CAM, NGFR, PIP5K1C), possibly reflecting the asymmetric autonomic innervation of the atrial chambers. Right atria were also enriched for proteins involved in natriuretic peptide signaling. To evaluate the role of our chamber specific findings on other cardiac phenotypes, we queried the phenotype wide association study (PheWAS), which is based on the UK-Biobank data of 408,961 white British European-ancestry samples representing thousands of phenotypes (17, 34). For proteins in the interaction network related to natriuretic peptide signaling we found that they were essentially all associated with hypertension (Fig. 5*C*). These data support the notion of the involvement of the right atrium in the humoral regulation of blood pressure.

## DISCUSSION

### 

#### 

##### First Comprehensive Map of the Chamber-specific Human Cardiac Proteome from Live Individuals

Important differences exist in the structure and function of the four cardiac chambers. Yet, differences in their molecular composition remain poorly studied. Here, we generated a detailed, quantitative map of >7000 proteins cataloged by chamber-specific expression. The samples, obtained from patients undergoing surgery for mitral valve replacement, were immediately snap-frozen to retain the physiological protein fingerprints of living human hearts. Our data showed improved quantitative quality compared with previous proteomics studies of human cardiac samples (7), which were affected by unspecific post-mortem protein degradation. We provide absolute protein quantification of all proteins within each chamber, as well as relative protein quantifications across chambers determined from mass spectrometry-based intensity measurements. We retrieved hundreds of chamber-specific molecular signatures and show that these meaningfully reflect the specialized functions and disease state of the respective chambers. To our knowledge, our dataset is first in identifying the differential proteome that can be obtained between a dilated LA and the non-dilated RA. Our dataset also greatly expands on the characterization of the differential atrial *versus* ventricle proteome and seeks correlation to chamber-specific disease states. However, important limitations of our study need to be considered.

##### Study Limitations

A comprehensive study of chamber-specific differences would require us to include samples from the right ventricle; yet, that cardiac region was not accessible through the surgical approach applied to the MVP patients. We do acknowledge that characterization of the differential RV *versus* LV proteome is a fundamental question of great potential impact; yet, as we show in our study, chamber-specific differences are best studied when the samples are collected from living individuals, even if at the expense of limits in the areas accessible to data collection.

The quantitative proteomes we present are covered at great depth, but we cannot be certain that our protein measurements covered the entire protein population because of limitation in instrument sensitivity. In that sense, absence of measurement of a protein in our experiments does not reflect an altogether absence of the protein in the measured samples.

Our measurements originate from tissue samples, which intrinsically bare more heterogeneity than *e.g.* cell lines. We ensured that blood contamination was not a confounding factor for protein quantification across cardiac chambers. Although some proteins can be attributed to certain cell types like cardiomyocytes, our data should be complemented with cell-specific measurements in the future to further differentiate the role of different cell types and their proteomes in cardiac function.

To characterize the proteome of the dilated left atria, we used the right atrial samples as reference. In doing so, we cannot be sure that all differences observed are consequent to the dilation of the tissue, rather than intrinsic to the native differences between the two atria. Although this question cannot be formally answered without studying samples from healthy individuals (which of course, faces obvious ethical limits), it is worth noting that the fraction of proteins with differential LA *versus* RA expression was less than 2% of the total and that in this differential, we identified molecules previously linked to remodeling processes known to occur in stressed tissue, namely, fibrosis, inflammation, and transcriptional modulation. It is appealing to evaluate the proteome of the dilated left atria from the point of view of relevance to atrial fibrillation. Inclusion of a single patient with atrial fibrillation has partially allowed for the comparison, but not in a quantitative manner. Furthermore, for an extensive evaluation of changes in a dilated left atrium in a pre-AF stage, it would be a necessity to stratify how risk factors such as age, sex, and hypertension would affect the results.

Although finding signs of a stressed tissue in the proteome of the LA was expected, the identity of the specific molecules that were differentially expressed was not predictable from the outset. Our study therefore carries its significance not only in identifying molecular groups that associate with a function, but also in singling out the specific elements of a network that deviate from the reference.

##### MVP and the Proteome

Our samples were obtained from patients with MVP and as such, we had the opportunity to study human heart proteome in the context of this disease. As a further step, we intersected our data with GWAS-based information on disease-associated loci (35). Indeed, an inherent difficulty of GWAS is to pinpoint the specific part of a locus that is causal of the phenotype, because each locus contains multiple genes. By intersecting existing genomic with proteomic data we not only corroborated gene associations for two molecules (LMCD1 and TNS1), but also yielded three new products (PITPNB, CBR1, and SMG6) as potentially linked to the disease. Our approach is not proof of involvement of these proteins in MVP; it is an additional way in which candidate genes in a genome-wide significant locus can be prioritized as potential participants in the resulting phenotype. Further studies will be necessary to corroborate this association.

##### The Atria Versus Ventricle Proteome

Quantification of proteins from the left ventricle allowed us to identify the molecular differences between the LV and the atria. Using the GTEx transcriptome database, we found that most of the differentially expressed proteins were also different at the transcript level. This strongly suggests that the differential is largely established because of gene regulation. The mechanisms of this regulation are not known, but likely involve mechanosensitive transcription factors. Furthermore, we found that molecules identified with familial cardiomyopathies that manifest primarily in the ventricle (such as desmosomal molecules in ARVC) were strongly over-represented in the ventricle. This indicates that over-expression is not a manifestation of redundancy but a requirement to maintain the physiological demand.

##### The Differential Proteome of a Dilated, Non-fibrillating LA

The association between left atrial dilation and AF is well established (36). Recent studies have also associated AF to fibrotic and inflammatory processes (37), as well as to transcriptional reprogramming (32) (see below). Yet, there is a paucity of evidence as to whether, in the human atria, these molecular changes precede, exist independent of, or are a consequence of the persistent fibrillation of the atria. Our data show that protein changes previously linked to persistent AF are actually present in the heart of patients with no evidence of persistent AF. These include PTX3, TGFB1 and TBX5. PTX3 represents a rapid biomarker for local innate immunity activation and inflammation, and atrial PTX3 concentrations were found to be higher in patients with persistent AF (37). TGFB1, on the other hand, stimulates the collagen-producing myofibroblasts thus contributing to cardiac fibrosis (38), and mRNA expression of TGFB1 has been found up-regulated in the atria of patients with permanent AF (32). Finally, TBX5, is a molecule associated with a gene regulatory network that maintains atrial rhythm (33). Its over-representation in the RA may signal a down-regulation in the LA, a modification that would be pro-AF, based on recent studies (33). Yet, although these three indicators of persistent AF were modified in the heart samples of our patients, persistent AF was not detected. As such, our data strongly indicate that these specific molecules, albeit linked to the disease, are not enough to support persistent AF. From that perspective, our study reveals the proteome of a left atrium either in the pre-AF, or in the non-AF stage and establishes a clear boundary between what is sufficient (or not) to obtain the persistent AF phenotype.

Though our studies did not directly document atrial fibrosis, we do document a pro-fibrotic molecular profile in the LA, consistent with the expected consequence of tissue dilation. More importantly, our study advances new knowledge by revealing the identity of the pro-fibrotic molecules present at this stage and as such, provides an entry point to a better understanding of the molecular steps and the possible biomarkers that can identify a pro-fibrotic, non-AF stage.

##### Conclusion and Outlook

We have generated the most comprehensive protein map of the heart from samples obtained *in vivo*. We show that the use of samples acquired *in vivo* (vis-à-vis post-mortem) greatly improves the quantitation of proteins across samples. We combine the power of proteomics with that of other big data sets to identify new gene candidates involved in MVP, to suggest that atria *versus* ventricle differences originate primarily at the level of gene regulation and to propose that excess abundance of proteins in the ventricle reflect not redundancy, but functional need. Most importantly, we provide the first proteome of human dilated LA in a non-AF state and show that increased levels of proteins previously associated with sustained AF are not enough to generate the disease state. Identification of chamber-specific differences can lead the way to the development of chamber-targeted therapeutic strategies.

## DATA AVAILABILITY

All MS raw data and search results from this study were uploaded to the ProteomeXchange Consortium via the PRIDE repository (13) with the identifier PXD008722 (accessible through https://www.ebi.ac.uk/pride/archive/login).

## Supplementary Material

Supplementary Material

Supplementary Table S1

Supplementary Table S2

Supplementary Table S3

Supplementary Table S4

## References

[1] Lindskog C., Linné J., Fagerberg L., Hallström B. M., Sundberg C. J., Lindholm M., Huss M., Kampf C., Choi H., Liem D. A., Ping P., Väremo L., Mardinoglu A., Nielsen J., Larsson E., Pontén F., and Uhlén M. (2015) The human cardiac and skeletal muscle proteomes defined by transcriptomics and antibody-based profiling. BMC Genomics 16, 475 26109061 10.1186/s12864-015-1686-y PMC4479346

[2] Kääb S., Barth A. S., Margerie D., Dugas M., Gebauer M., Zwermann L., Merk S., Pfeufer A., Steinmeyer K., Bleich M., Kreuzer E., Steinbeck G., and Näbauer M. (2004) Global gene expression in human myocardium—oligonucleotide microarray analysis of regional diversity and transcriptional regulation in heart failure. J. Mol. Med. 82, 308–316 15103417 10.1007/s00109-004-0527-2

[3] Wilhelm M., Schlegl J., Hahne H., Moghaddas Gholami A., Lieberenz M., Savitski M. M., Ziegler E., Butzmann L., Gessulat S., Marx H., Mathieson T., Lemeer S., Schnatbaum K., Reimer U., Wenschuh H., Mollenhauer M., Slotta-Huspenina J., Boese J.-H., Bantscheff M., Gerstmair A., Faerber F., and Kuster B. (2014) Mass-spectrometry-based draft of the human proteome. Nature 509, 582–587 24870543 10.1038/nature13319

[4] Kim M.-S., Pinto S. M., Getnet D., Nirujogi R. S., Manda S. S., Chaerkady R., Madugundu A. K., Kelkar D. S., Isserlin R., Jain S., Thomas J. K., Muthusamy B., Leal-Rojas P., Kumar P., Sahasrabuddhe N. A., Balakrishnan L., Advani J., George B., Renuse S., Selvan L. D. N., Patil A. H., Nanjappa V., Radhakrishnan A., Prasad S., Subbannayya T., Raju R., Kumar M., Sreenivasamurthy S. K., Marimuthu A., Sathe G. J., Chavan S., Datta K. K., Subbannayya Y., Sahu A., Yelamanchi S. D., Jayaram S., Rajagopalan P., Sharma J., Murthy K. R., Syed N., Goel R., Khan A. A., Ahmad S., Dey G., Mudgal K., Chatterjee A., Huang T.-C., Zhong J., Wu X., Shaw P. G., Freed D., Zahari M. S., Mukherjee K. K., Shankar S., Mahadevan A., Lam H., Mitchell C. J., Shankar S. K., Satishchandra P., Schroeder J. T., Sirdeshmukh R., Maitra A., Leach S. D., Drake C. G., Halushka M. K., Prasad T. S. K., Hruban R. H., Kerr C. L., Bader G. D., Iacobuzio-Donahue C. A., Gowda H., and Pandey A. (2014) A draft map of the human proteome. Nature 509, 575–581 24870542 10.1038/nature13302 PMC4403737

[5] Aye T. T., Scholten A., Taouatas N., Varro A., Van Veen T. a. B., Vos M. a., and Heck A. J. R. (2010) Proteome-wide protein concentrations in the human heart. Mol. BioSystems 6, 1917 10.1039/c004495d 20596566

[6] Lu Z. Q., Sinha A., Sharma P., Kislinger T., and Gramolini A. O. (2014) Proteomic analysis of human fetal atria and ventricle. J. Proteome Res. 13, 5869–5878 25323733 10.1021/pr5007685

[7] Doll S., Dreβen M., Geyer P. E., Itzhak D. N., Braun C., Doppler S. A., Meier F., Deutsch M.-A., Lahm H., Lange R., Krane M., and Mann M. (2017) Region and cell-type resolved quantitative proteomic map of the human heart. Nat. Communications 8 10.1038/s41467-017-01747-2 PMC5684139 29133944

[8] Tyanova S., Temu T., Sinitcyn P., Carlson A., Hein M. Y., Geiger T., Mann M., and Cox J. (2016) The Perseus computational platform for comprehensive analysis of (prote)omics data. Nat. Methods 13, 731–740 27348712 10.1038/nmeth.3901

[9] Tusher V. G., Tibshirani R., and Chu G. (2001) Significance analysis of microarrays applied to the ionizing radiation response. Proc. Natl. Acad. Sci. U.S.A. 98, 5116–5121 11309499 10.1073/pnas.091062498 PMC33173

[10] Bekker-Jensen D. B., Kelstrup C. D., Batth T. S., Larsen S. C., Haldrup C., Bramsen J. B., Sørensen K. D., Høyer S., Ørntoft T. F., Andersen C. L., Nielsen M. L., and Olsen J. V. (2017) An optimized shotgun strategy for the rapid generation of comprehensive human proteomes. Cell Systems 4, 587–599.e584 28601559 10.1016/j.cels.2017.05.009 PMC5493283

[11] Tyanova S., Temu T., and Cox J. (2016) The MaxQuant computational platform for mass spectrometry-based shotgun proteomics. Nat. Protocols 11, 2301–2319 27809316 10.1038/nprot.2016.136

[12] Bolstad B. M., Irizarry R. A., Astrand M., and Speed T. P. (2003) A comparison of normalization methods for high density oligonucleotide array data based on variance and bias. Bioinformatics 19, 185–193 12538238 10.1093/bioinformatics/19.2.185

[13] Vizcaíno J. A., Csordas A., del-Toro N., Dianes J. A., Griss J., Lavidas I., Mayer G., Perez-Riverol Y., Reisinger F., Ternent T., Xu Q.-W., Wang R., and Hermjakob H. (2016) 2016 update of the PRIDE database and its related tools. Nucleic Acids Res. 44, D447–D456 26527722 10.1093/nar/gkv1145 PMC4702828

[14] Batth T. S., Francavilla C., and Olsen J. V. (2014) Off-line high-pH reversed-phase fractionation for in-depth phosphoproteomics. J. Proteome Res. 13, 6176–6186 25338131 10.1021/pr500893m

[15] Dina C., Bouatia-Naji N., Tucker N., Delling F. N., Toomer K., Durst R., Perrocheau M., Fernandez-Friera L., Solis J., Investigators P., Le Tourneau T., Chen M.-H., Probst V., Bosse Y., Pibarot P., Zelenika D., Lathrop M., Hercberg S., Roussel R., Benjamin E. J., Bonnet F., Lo S. H., Dolmatova E., Simonet F., Lecointe S., Kyndt F., Redon R., Le Marec H., Froguel P., Ellinor P. T., Vasan R. S., Bruneval P., Markwald R. R., Norris R. A., Milan D. J., Slaugenhaupt S. A., Levine R. A., Schott J.-J., Hagege A. A., MVP-France Jeunemaitre X., and Network L. T. M. (2015) Genetic association analyses highlight biological pathways underlying mitral valve prolapse. Nat. Genet. 47, 1206 26301497 10.1038/ng.3383 PMC4773907

[16] Schwanhäusser B., Busse D., Li N., Dittmar G., Schuchhardt J., Wolf J., Chen W., and Selbach M. (2011) Global quantification of mammalian gene expression control. Nature 473, 337–342 21593866 10.1038/nature10098

[17] Bycroft C., Freeman C., Petkova D., Band G., Elliott L. T., Sharp K., Motyer A., Vukcevic D., Delaneau O., O'Connell J., Cortes A., Welsh S., McVean G., Leslie S., Donnelly P., and Marchini J. (2017) Genome-wide genetic data on ∼500,000 UK Biobank participants. bioRxiv

[18] Small E. M., and Krieg P. A. (2004) Molecular Regulation of Cardiac Chamber-Specific Gene Expression. Trends Cardiovascular Med. 14, 13–18 10.1016/j.tcm.2003.09.005 14720469

[19] Hailstones D., Barton P., Thomas P., Sasse S., Sutherland C., Hardeman E., and Gunning P. (1992) Differential regulation of the atrial isoforms of the myosin light chains during striated muscle development. J. Biol. Chem. 267, 23295–23300 1429676

[20] Krupnik V. E., Sharp J. D., Jiang C., Robison K., Chickering T. W., Amaravadi L., Brown D. E., Guyot D., Mays G., Leiby K., Chang B., Duong T., Goodearl A. D., Gearing D. P., Sokol S. Y., and McCarthy S. A. (1999) Functional and structural diversity of the human Dickkopf gene family. Gene 238, 301–313 10570958 10.1016/s0378-1119(99)00365-0

[21] Monaghan A. P., Kioschis P., Wu W., Zuniga A., Bock D., Poustka A., Delius H., and Niehrs C. (1999) Dickkopf genes are co-ordinately expressed in mesodermal lineages. Mech. Dev. 87, 45–56 10495270 10.1016/s0925-4773(99)00138-0

[22] Barefield D. Y., Puckelwartz M. J., Kim E. Y., Wilsbacher L. D., Vo A. H., Waters E. A., Earley J. U., Hadhazy M., Dellefave-Castillo L., Pesce L. L., and McNally E. M. (2017) Experimental modeling supports a role for MyBP-HL as a novel myofilament component in arrhythmia and dilated cardiomyopathy. Circulation 136, 1477–1491 28778945 10.1161/CIRCULATIONAHA.117.028585 PMC5645234

[23] Shannon P., Markiel A., Ozier O., Baliga N. S., Wang J. T., Ramage D., Amin N., Schwikowski B., and Ideker T. (2003) Cytoscape: a software environment for integrated models of biomolecular interaction networks. Genome Res. 13, 2498–2504 14597658 10.1101/gr.1239303 PMC403769

[24] Morris J. H., Apeltsin L., Newman A. M., Baumbach J., Wittkop T., Su G., Bader G. D., and Ferrin T. E. (2011) clusterMaker: a multi-algorithm clustering plugin for Cytoscape. BMC Bioinformatics 12, 436 22070249 10.1186/1471-2105-12-436 PMC3262844

[25] Szklarczyk D., Morris J. H., Cook H., Kuhn M., Wyder S., Simonovic M., Santos A., Doncheva N. T., Roth A., Bork P., Jensen L. J., and von Mering C. (2017) The STRING database in 2017: quality-controlled protein–protein association networks, made broadly accessible. Nucleic Acids Res. 45, D362–D368 27924014 10.1093/nar/gkw937 PMC5210637

[26] Lonsdale J., Thomas J., Salvatore M., Phillips R., Lo E., Shad S., Hasz R., Walters G., Garcia F., Young N., Foster B., Moser M., Karasik E., Gillard B., Ramsey K., Sullivan S., Bridge J., Magazine H., Syron J., Fleming J., Siminoff L., Traino H., Mosavel M., Barker L., Jewell S., Rohrer D., Maxim D., Filkins D., Harbach P., Cortadillo E., Berghuis B., Turner L., Hudson E., Feenstra K., Sobin L., Robb J., Branton P., Korzeniewski G., Shive C., Tabor D., Qi L., Groch K., Nampally S., Buia S., Zimmerman A., Smith A., Burges R., Robinson K., Valentino K., Bradbury D., Cosentino M., Diaz-Mayoral N., Kennedy M., Engel T., Williams P., Erickson K., Ardlie K., Winckler W., Getz G., DeLuca D., MacArthur D., Kellis M., Thomson A., Young T., Gelfand E., Donovan M., Meng Y., Grant G., Mash D., Marcus Y., Basile M., Liu J., Zhu J., Tu Z., Cox N. J., Nicolae D. L., Gamazon E. R., Im H. K., Konkashbaev A., Pritchard J., Stevens M., Flutre T., Wen X., Dermitzakis E. T., Lappalainen T., Guigo R., Monlong J., Sammeth M., Koller D., Battle A., Mostafavi S., McCarthy M., Rivas M., Maller J., Rusyn I., Nobel A., Wright F., Shabalin A., Feolo M., Sharopova N., Sturcke A., Paschal J., Anderson J. M., Wilder E. L., Derr L. K., Green E. D., Struewing J. P., Temple G., Volpi S., Boyer J. T., Thomson E. J., Guyer M. S., Ng C., Abdallah A., Colantuoni D., Insel T. R., Koester S. E., Little A. R., Bender P. K., Lehner T., Yao Y., Compton C. C., Vaught J. B., Sawyer S., Lockhart N. C., Demchok J., and Moore H. F. (2013) The Genotype-Tissue Expression (GTEx) project. Nat. Genet. 45, 580 23715323 10.1038/ng.2653 PMC4010069

[27] Kahr P. C., Piccini I., Fabritz L., Greber B., Scholer H., Scheld H. H., Hoffmeier A., Brown N. A., and Kirchhof P. (2011) Systematic analysis of gene expression differences between left and right atria in different mouse strains and in human atrial tissue. PLoS ONE 6, e26389 22039477 10.1371/journal.pone.0026389 PMC3198471

[28] Padang R., Bagnall R. D., and Semsarian C. (2012) Genetic basis of familial valvular heart disease. Circ. Cardiovasc. Genet. 5, 569–580 23074336 10.1161/CIRCGENETICS.112.962894

[29] Asp J., Synnergren J., Jonsson M., Dellgren G., and Jeppsson A. (2012) Comparison of human cardiac gene expression profiles in paired samples of right atrium and left ventricle collected in vivo. Physiol. Genomics 44, 89–98 22085905 10.1152/physiolgenomics.00137.2011

[30] Frolova E. G., Sopko N., Blech L., Popovic Z. B., Li J., Vasanji A., Drumm C., Krukovets I., Jain M. K., Penn M. S., Plow E. F., and Stenina O. I. (2012) Thrombospondin-4 regulates fibrosis and remodeling of the myocardium in response to pressure overload. FASEB J. 26, 2363–2373 22362893 10.1096/fj.11-190728 PMC3360147

[31] Cingolani O. H., Kirk J. A., Seo K., Koitabashi N., Lee D. I., Ramirez-Correa G., Bedja D., Barth A. S., Moens A. L., and Kass D. A. (2011) Thrombospondin-4 is required for stretch-mediated contractility augmentation in cardiac muscle. Circ. Res. 109, 1410–1414 22034490 10.1161/CIRCRESAHA.111.256743 PMC3324097

[32] Barth A. S. (2005) Reprogramming of the human atrial transcriptome in permanent atrial fibrillation: expression of a ventricular-like genomic signature. Circ. Res. 96, 1022–1029 15817885 10.1161/01.RES.0000165480.82737.33

[33] Nadadur R. D., Broman M. T., Boukens B., Mazurek S. R., Yang X., van den Boogaard M., Bekeny J., Gadek M., Ward T., Zhang M., Qiao Y., Martin J. F., Seidman C. E., Seidman J., Christoffels V., Efimov I. R., McNally E. M., Weber C. R., and Moskowitz I. P. (2016) Pitx2 modulates a Tbx5-dependent gene regulatory network to maintain atrial rhythm. Sci. Transl. Med. 8, 354ra115 10.1126/scitranslmed.aaf4891 PMC5266594 27582060

[34] Zhou W., Nielsen J. B., Fritsche L. G., Dey R., Elvestad M. B., Wolford B. N., LeFaive J., VandeHaar P., Gifford A., Bastarache L. A., Wei W.-Q., Denny J. C., Lin M., Hveem K., Kang H. M., Abecasis G. R., Willer C. J., and Lee S. (2017) Efficiently controlling for case-control imbalance and sample relatedness in large-scale genetic association studies. bioRxiv10.1038/s41588-018-0184-y PMC6119127 30104761

[35] Lundby A., Rossin E. J., Steffensen A. B., Acha M. R., Newton-Cheh C., Pfeufer A., Lynch S. N., Olesen S.-P., Brunak S., Ellinor P. T., Jukema J. W., Trompet S., Ford I., Macfarlane P. W., Krijthe B. P., Hofman A., Uitterlinden A. G., Stricker B. H., Nathoe H. M., Spiering W., Daly M. J., Asselbergs F. W., van der Harst P., Milan D. J., de Bakker P. I. W., Lage K., and Olsen J. V. (2014) Annotation of loci from genome-wide association studies using tissue-specific quantitative interaction proteomics. Nat. Methods 11, 868–874 24952909 10.1038/nmeth.2997 PMC4117722

[36] Hu Y.-F., Chen Y.-J., Lin Y.-J., and Chen S.-A. (2015) Inflammation and the pathogenesis of atrial fibrillation. Nat. Rev. Cardiol. 12, 230 25622848 10.1038/nrcardio.2015.2

[37] Soeki T., Bando S., Uematsu E., Matsuura T., Niki T., Ise T., Kusunose K., Hotchi J., Ueda Y., Tomita N., Yamaguchi K., Yagi S., Fukuda D., Taketani Y., Iwase T., Yamada H., Wakatsuki T., Shimabukuro M., and Sata M. (2014) Pentraxin 3 is a local inflammatory marker in atrial fibrillation. Heart Vessels 29, 653–658 23979265 10.1007/s00380-013-0400-8

[38] Akiyama-Uchida Y., Ashizawa N., Ohtsuru A., Seto S., Tsukazaki T., Kikuchi H., Yamashita S., and Yano K. (2002) Norepinephrine enhances fibrosis mediated by TGF-beta in cardiac fibroblasts. Hypertension 40, 148–154 12154105 10.1161/01.hyp.0000025443.61926.12

