# Supplementary material for: Quantitative Proteomics of Human Heart Samples Collected In Vivo Reveal the Remodeled Protein Landscape of Dilated Left Atrium Without Atrial Fibrillation
Source: Mol Cell Proteomics. 2020 Apr 14;19(7):1132–44. doi: 10.1074/mcp.RA119.001878 (PMC7338087; doi:10.1074/mcp.RA119.001878)
Supplement: Supplementary Material [file 157217_1_supp_489907_q74rxm.pdf]

Supplementary Material

Contents

Contents 1

Material and Methods 3

Experimental Design and Statistical Rationale

Tissue collection 3

Tissue Homogenization 3

Peptide preparation 4

Offline high pH fractionation of peptide samples 4

LC-MS/MS measurements 5

Data analysis 5

Raw data processing in MaxQuant 5

Quantile normalization of raw intensity data 6

Principal Component Analysis 7

Hierarchical clustering 7

Outlier identification 7

Volcano Plot Analysis 7

Enrichment analysis of significant proteins 8

Comparison of protein abundance and RNA expression data from the GTEx consortium 8

Analysis of proteins associated with dilated cardiomyopathy 9

Data availability 10

Supplementary Figures 12
Supplementary Figure S1 12
Supplementary Figure S2 14
Supplementary Figure S3 15
Supplementary Figure S4 20
Supplementary Figure S5 23
Supplementary Figure S6 25
Supplementary Figure S7 29
Supplementary Figure S8 27
Supplementary Tables 30
References 32

Word Count Supplementary: 4655

Material and Methods

Experimental Design and Statistical Rationale

To ensure high quality data for a majority of identified proteins biopsies were included from seven patients.

Tissue collection

Cardiac tissue biopsies were collected from the endocardial side of the heart extending several millimeters into the myocardium. Specifically, biopsies were collected from the lateral wall of the right atrium, the posterior wall of the left atrium and from a papillary muscle in the left ventricle of seven patients undergoing mitral valve surgery. The tissue samples were collected by the surgeon during surgery just prior to the valve plasty or removal and immediately transferred to sterilized tubes to be snap-frozen in a container with liquid nitrogen while still in the operating room.

Tissue Homogenization

Frozen tissue biopsies were homogenized on a Precellys24 homogenizer (Bertin Technologies, France) in tissue incubation buffer (50mM Tris-HCl pH 8.5, 5mM EDTA, 150mM NaCl, 10mM KCl, 1% Triton X-100, 5mM NaF, 5mM beta-glycerophosphate, 1mM Na-orthovanadate, containing 1x Roche complete protease inhibitor) with ceramic beads (2.8 and 1.4mm zirconium oxide beads, Precellys). Homogenates were incubated for 2h at 4°C (20rpm), centrifuged (15000g, 20min, 4°C) and soluble fractions transferred to chilled 1.5mL tubes.

To remove surfactants prior to LC-MS/MS measurement, protein was precipitated by addition of acetone (at ratio 5:1 v/v), 1h incubation at -20°C and gentle centrifugation (400g, 1.5min). Supernatants were discarded and protein resuspended in Guanidine-HCl buffer (Gnd-HCl; 6MGnd-HCl, 50mM Tris HCl pH 8.5, 5mM NaF, 5mM beta-glycerophosphate, 1mM Na-orthovanadate, containing 1x Roche complete protease inhibitor). Disulfide bridges were reduced and cysteine moieties alkylated by addition of 5mM Tris(2-

carboxyethyl)phosphine (TCEP) and 10mM chloroacetamide (CAA) and incubation in the dark at room temperature for 15min.

Peptide preparation

From each sample, up to 1mg protein was digested in-solution by addition of endoproteinase Lys-C (Trichem ApS, Denmark; 1:100 enzyme:protein ratio) for 1.5h at 30°C, 750rpm in the dark, followed by dilution (1:12 with 50mM Tris-HCl pH8) and digestion with trypsin overnight (14h) at 37°C, 750rpm (Life technologies, USA, 1:100 enzyme:protein ratio). The reactions were quenched by addition of trifluoroacetic acid (TFA, 1% final conc.) and samples were centrifuged (14000g, 10min) to sediment debris. Soluble fractions were desalted and concentrated on C18 SepPak columns (Waters, USA). Peptides were eluted with 40% acetonitirle (ACN) followed by 60% ACN, and organic solvents subsequently evaporated by vacuum centrifugation.

Offline high pH fractionation of peptide samples

Of each sample, 50-100ug peptide (in 10uL injection volume) was fractionated by micro-flow reverse-phase ultrahigh pressure liquid chromatograpy (UPLC) on an Dionex UltiMate 3000 UPLC system (Thermo Scientific, USA) equipped with an ACQUITY UPLC CSH C18 Column (130Å, 1.7 µm, 1 mm X 150 mm) at 30µL/min flow rate essentially as previously described [1]. The following 85min gradient elution program was used employing a binary pump connected to Solvent A (5mM ammonium bicarbonate (ABC), pH8) and B (100% ACN): 0-50 min: 4.5-22.5% B, 50-55 min: 22.5-63% B, 55-60 min: 63% B isocratic, 60-62 min: 63-81% B, 62-70 min: 81% B isocratic, 70-75 min: 81-4.5% B, followed by column re-equilibration at 4.5% B for 10 min. Outflow from 4-60 minutes was collected in 1-minute intervals into 12 concatenated fractions in the autosampler. Fractions were acidified by addition of 5µL 5% formic acid (FA) to avoid precipitation of ABC, and fraction volume was reduced by vacuum centrifugation.

If not specified otherwise, chemicals and reagents were acquired from Sigma Aldrich, USA. Chromatography solvents were acquired from VWR, USA.

LC-MS/MS measurements

Fractionated peptide samples were analyzed by online reversed-phase liquid chromatography coupled to a Q-Exactive HF quadrupole Orbitrap tandem mass spectrometer (LC-MS/MS, Thermo Electron, Bremen, Germnay). Peptide samples were brought to concentration of 0.2ug/uL (diluted in 5%ACN, 0.1% TFA) in 96-well microtiter plates and autosampled (5uL injection volume) into a nanoflow Easy-nLC system (Proxeon Biosystems, Odense, Denmark). Peptide samples were separated on 15cm fused-silica emitter columns pulled and packed in-house with reversed-phase ReproSil-Pur C18-AQ 1.9µm resin (Dr. Maisch GmbH, Ammerbuch-Entringen, Germany) in a 1h multi-step linear gradient (0.1% formic acid constant; 2-25%ACN in 45min, 25-45%ACN in 8min, 45-80%ACN in 3 min) followed by short column re-equilibration (80-5%ACN in 5min, 5%ACN for 2min). Column effluent was directly ionized in a nano-electrospray ionization source operated in positive ionization mode and electrosprayed into the mass spectrometer.

Full-MS spectra (375-1500 m/z) were acquired after accumulation of 3,000,000 ions in the Orbitrap (maximum fill time of 25ms) at 120,000 resolution. A data-dependent Top12 method then sequentially isolated the most intense precursor ions (up to 12 per full scan) for higher-energy collisional dissociation (HCD) in an octopole collision cell. MS/MS spectra of fragment ions were recorded at resolution of 30000 after accumulation of 100,000 ions in the Orbitrap (maximum fill time of 45ms).

Data analysis

Raw data processing in MaxQuant

Raw MS data was processed using the MaxQuant software [2] version 1.5.3.30 (Max-Planck Institute of Biochemistry, Department of Proteomics and Signal Transduction, Munich) and proteins identified with the built-in Andromeda search engine by searching MS/MS spectra

against an in-silico tryptic digest of a database containing all reviewed human SwissProt protein entries including protein isoforms (42138 protein entries, downloaded on 7.3.2016). Raw files originating from fractionated samples of the same tissue biopsy were grouped for the search (resulting in 7 biological replicates for 3 cardiac chambers). The MS/MS spectra were searched with Carbamidomethly-Cysteine as fixed modification, as well as oxidation (M), acetylation of protein N-termini and Gln->pyro-Glu as variable modifications. Of these variable modifications, a maximum of six were allowed per peptide. Also, a maximum of two missed cleavages was allowed per peptide. The minimum peptide length was set to 7 amino acids (default) and minimum Andromeda score required for modified peptides was 25, with minimum delta score of 6 (default). First search tolerance was 20ppm (default) and main search tolerance was 4.5ppm (default), requiring strict specificity of tryptic peptides. Due to the similarity of the samples the match-between-runs option was enabled with default parameters. False-discovery rate cutoffs were set to 1% on peptide, protein and site decoy level (default), only allowing high-quality identifications to pass. One scrambled decoy peptide was included in the reverse decoy database for each theoretically possible true peptide in the forward database. All peptides were used for protein quantification, and label-free quantification (LFQ) was performed in MaxQuant with fast LFQ option enabled.

Protein identification results were further processed using the Perseus software suite [3] version 1.5.5.3.

Quantile normalization of raw intensity data

In order to remove minor technical variation between samples, quantile normalization of raw intensities was performed based on the Bioconductor R package LIMMA [4]. For quantitative analysis, protein groups representing isoforms of the same canonical protein were removed from the data such that only the most abundant protein isoform was retained (6887 protein groups remained).

Principal Component Analysis

Normalized raw data was log_{10}-transformed and filtered for 100% valid values and Principal Component Analysis (PCA) was performed in Perseus on the remaining 4043 proteins.

Hierarchical clustering

Normalized raw intensity values were log_{10}-transformed and filtered for proteins identified in at least 67% in at least one chamber (valid values in 5/7 or 4/6 replicates; 6007 protein groups remained). Unsupervised hierarchical clustering was performed in Perseus using standard parameters (Euclidean distance with average linkage, 300 clusters, 10 iterations, 1 restart, and k-means preprocessing for both row and column tree).

Outlier identification

Sample H3-LA was consistently found to be an outlier by both principal component analysis (PCA) and hierarchical clustering (data not show). This finding can be readily explained by below-average biopsy size and very low protein yield observed in the original sample. H3-LA was thus excluded from all further analyses, and PCA and Hierarchical clustering repeated with the remaining 20 samples.

Volcano Plot Analysis

Normalized raw intensity values were log_2-transformed and filtered for proteins identified in at least 67% in at least one chamber (valid values in 5/7 or 4/6 replicates). Missing values were imputed using standard parameters in Perseus (width 0.3, down-shift 1.8) and proteins with <3 identified peptides were excluded from further analyses (5707 protein groups remained). Volcano Plot analysis was performed in Perseus (Student's T-test, S0=0.1, 5% permutation-based FDR with 750 iterations). In the volcano plot, protein intensity fold-change between tissues is represented as log_2 (difference of mean protein intensity). We subsequently manually checked all significant proteins for artifacts of imputation and removed significant proteins for which imputation was the only reason for significant

difference between chambers (5688 protein groups remained in volcano plot V vs. A, no proteins removed in RA vs. LA).

Enrichment analysis of significant proteins

Overrepresentation analysis for Pathway and Gene Ontology (GO) terms was performed on sets of significant proteins from volcano plot analysis. Enrichment analysis for all significant proteins was performed in InnateDB (http://www.innatedb.ca/). For a more refined overrepresentation analysis, we subsequently retrieved protein association networks of significant proteins from STRING using the STRING App (v.1.1.1) in Cytoscape (v.3.4.0), performed MCL clustering (clusterMaker2 v.1.1.0) of the network relative to association score, and retrieved enriched terms (GOBP, GOCC, GOMP, and KEGG) of highly interconnected protein clusters with a p-value cutoff of 0.05 using the STRING App [5-7].

Comparison of protein abundance and RNA expression data from the GTEx consortium

With the aim of assessing the correlation between RNA transcript level and protein abundance found in this study, transcriptomic data from 190 individuals was downloaded from the Genotype-Tissue Expression (GTEx) consortium portal (https://gtexportal.org/, V7 release). Specifically, we downloaded the median RNA-seq expression for left ventricle and atrial appendage ("GTEx_Analysis_2016-01-15_v7_RNASeQCv1.1.8_gene_median_tpm.gct.gz", downloaded on 22.09.2017). Data analysis was performed in Perseus (v1.5.5.3). Data was $\log_{10}$ transformed and transcript sample distributions for atria and ventricle were normalized by median subtraction. Normalized protein intensities from our study were $\log_{10}$ transformed and the median protein intensity was calculated across all left ventricle as well as all atrial samples. Proteome and transcriptome data was joined by gene name and plotted against each other (Sup. Fig. S2A+B). Fold-change between ventricle and atria was calculated from $\log_2$ transformed data for both proteome and transcriptome, plotted against each other and significant proteins from

our study were highlighted (Sup. Fig. S2C). The comparison between measured protein intensities and transcript information acquired from the GeTex database was based on 6,788 of the 7,314 proteins reported herein. The 6,788 proteins were the ones that could be matched to the GeTex dataset by their gene name.

Analysis of proteins associated with dilated cardiomyopathy

In order to assess protein expression of genetic associations to dilated cardiomyopathy (DCM), we extracted data on genetic mutations from Nouhravesh *et al*, 2016 [8]. To assess likelihood of true DCM association, Nouhravesh and colleagues tested DCM associated variants from the Human Gene Mutation Database (HGMD) [9] against prevalence of the same variant in a general population, assessed through the Exome Aggregation Consortium (ExAC) resource database [10]. A confidence score was calculated for each gene x by multiplying the number of variants found exclusively in HGMD (not in ExAC) with their fraction of the total no of variants found in HGMD, so that both high number of HGMD-exclusive mutations, as well as a high percentage of HGMD-exclusive vs. total mutations found weighted positively:

Confidence score $c_x = v_x * p_x$, where

Number of HGMD-exclusive variants of x v_x = Number of variants in $HGMD_x$ – Number of variants in $ExAC_x$

Fraction of HGMD-exclusive variants of x p_x = (Number of variants in $HGMD_x$ – Number of variants in $ExAC_x$) / Number of variants in $ExAC_x$

We then binned the DCM-associated genes into three categories of high, medium and low confidence of true disease association, where $c > 1$ corresponded to high confidence, and $c < 0.25$ to low confidence. This binning coincided with association of high confidence for genes with 3 or more HGMD-exclusive mutations, and low confidence assigned to genes with zero HGMD-exclusive mutations.

Based on this binned classification, we tested for significant difference in protein median intensities in left ventricle between the high and low confidence identifications (two-tailed Student's T-test). We found that genes in the low confidence bin showed significantly lower protein expression in left ventricle than the genes in the high confidence category ($p = 0.03$).

Data availability

All MS raw data and search results from this study were uploaded to the ProteomeXchange Consortium via the PRIDE repository [11] with the identifier PXD008722 (accessible through https://www.ebi.ac.uk/pride/archive/login, username: reviewer95074@ebi.ac.uk, password: 2IqsrqEy). Original sample names were simplified for publication according to the following scheme:

Sample name	Publication name
H097-LA	H1-LA
H097-LV	H1-LV
H097-RA	H1-RA
H113-LA	H2-LA
H113-LV	H2-LV
H113-RA	H2-RA
H117-LA	H3-LA
H117-LV	H3-LV
H117-RA	H3-RA
H119-LA	H4-LA
H119-LV	H4-LV
H119-RA	H4-RA
H141-LA	H5-LA
H141-LV	H5-LV
H141-RA	H5-RA
H145-LA	H6-LA
H145-LV	H6-LV
H145-RA	H6-RA
H148-LA	H7-LA
H148-LV	H7-LV
H148-RA	H7-RA

Supplementary Figures

Supplementary Figure S1

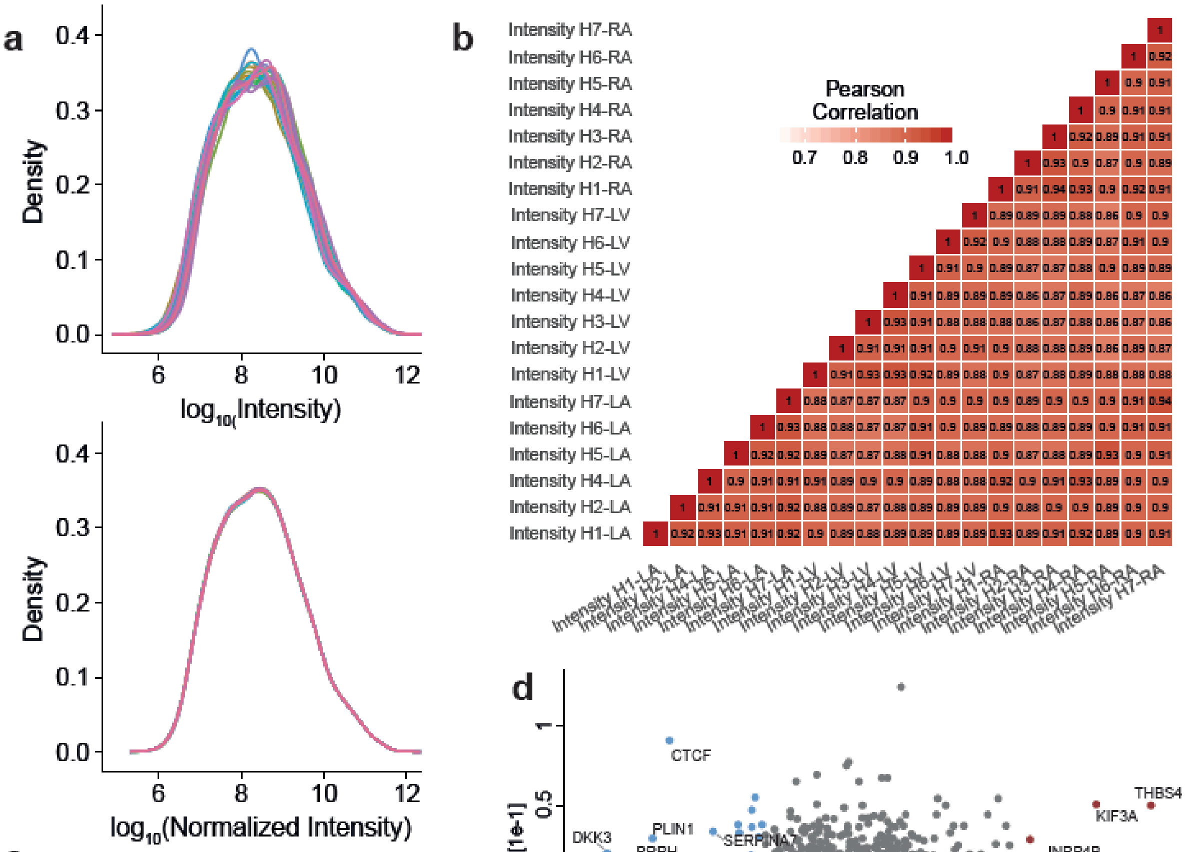

c

Left Atrium - Proteins in x / 6 replicates	#	% of total
6	4591	69
5	603	9
4	466	7
3	365	5
2	353	5
1	303	5
Proteins in at least 3 / 6 replicates	6025	90
Total	6681	100

Right Atrium - Proteins in x / 7 replicates	#	% of total
7	4842	71
6	584	9
5	361	5
4	351	5
3	246	4
2	215	3
1	189	3
Proteins in at least 4 / 7 replicates	6138	90
total	6788	100

Left Ventricle - Proteins in x / 7 replicates	#	% of total
7	4425	67
6	574	9
5	391	6
4	348	5
3	349	5
2	291	4
1	269	4
Proteins in at least 4 / 7 replicates	5738	86
Total	6647	100

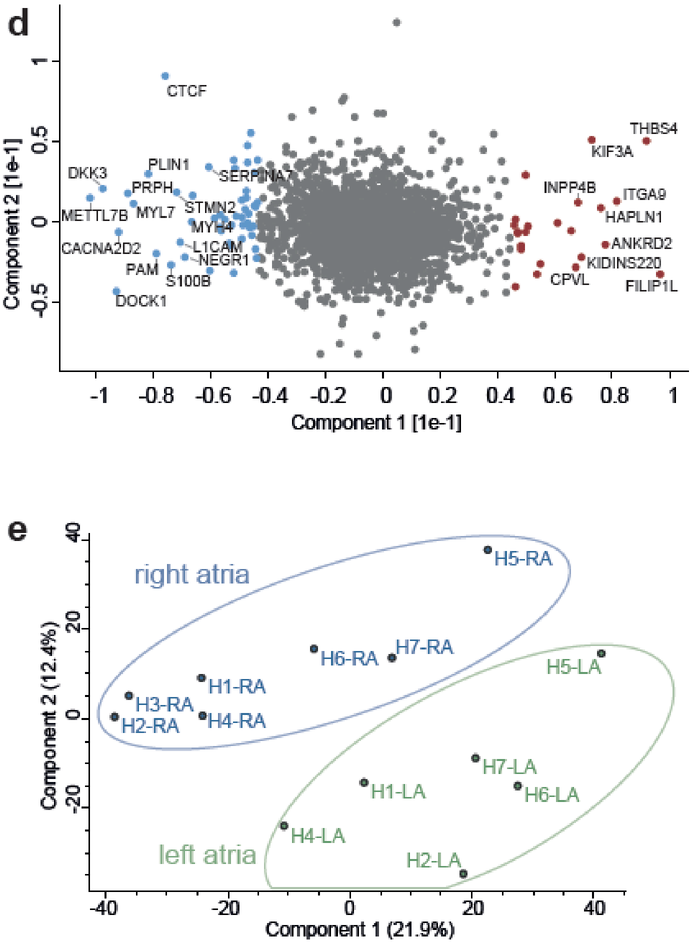

Supplementary Figure S1: Quality control of MS-based cardiac proteome data shows high biological and technical reproducibility. a. Quantile normalization (lower panel) of raw intensity distributions (upper panel) was performed to remove minor technical variation from the dataset **b.** Correlations of protein raw intensities between replicates within and across chambers are displayed as Pearson correlation coefficients for protein intensities across samples. H1 through H7 denotes the individuals in the study, RA, LV and LA denotes right atria, left ventricle and left atria, respectively **c.** Number of proteins identified in each chamber (LA, RA, LV) split into details of in how many samples the protein was identified in. Ie, for the right atrium 4842 proteins were identified in all seven samples, whereas 584 proteins were only identified in six of the seven samples, etc. In each chamber 86-90% of all proteins were identified in at least 50% of replicates **d**. Loadings corresponding to PCA of all samples (displayed in Fig. 1c) shows main drivers of differences between atrial (blue) and ventricular (red) samples **e**. Principal Component Analysis of only atrial samples showed clear distinction of right (blue) and left (green) atria along the two first components.

Supplementary Figure S2

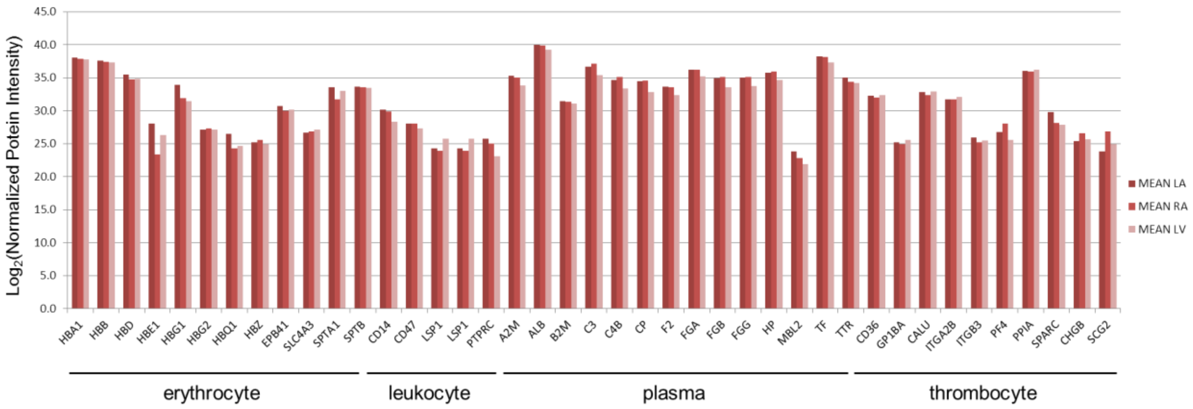

Supplementary Figure S2: Mean normalized protein intensities of selected blood proteins originating from erythrocytes, leukocytes, plasma and thrombocytes show overall similar protein intensity across chambers.

Supplementary Figure S3

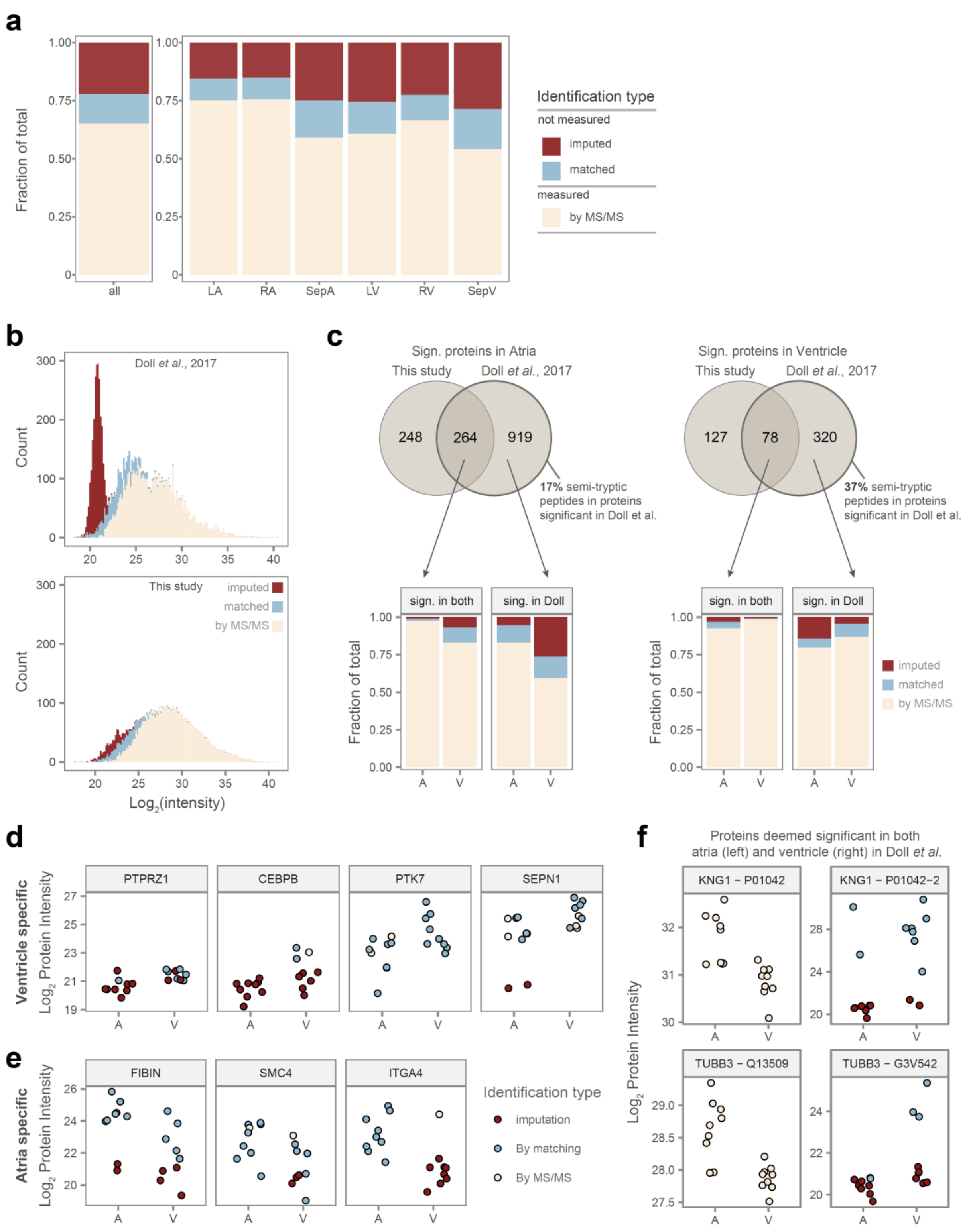

Supplementary Figure S3: Re-analysis of data from Doll *et al*. 2017 and comparison of results. a. Summary of protein identification type for data underlying volcano plot analysis in atria (LA, RA, SepA) versus ventricle (LV, RV or SepV) by Doll *et al.* [12]. In this part of their

analysis, ~30% of data points were not backed by MS/MS evidence; these data points were either only matched on MS1 level (blue) or values were imputed (red, e. g. missing values not detected in some replicates of the same tissue, or detected only in other cardiac regions). **b.** Representative examples of protein intensity distributions of a sample from Doll *et al.* (top, sample LV3) and from our study (bottom, sample H1-LV). In the histograms of protein intensities, protein identification based on MS/MS events are colored in beige, those based on match between runs are colored blue and imputed intensities are colored red. Upper panel: inclusion of ~30% missing values combined with an imputation strategy based on a narrow distribution lead to deviation of the data from a normal distribution. In comparison, imputation and matching is low in our dataset (lower panel) **c.** Venn diagrams showing the overlap of proteins deemed significant for either atria (left) or ventricle (right) in this study and the study by Doll *et al.* A considerably higher fraction of semi-tryptic peptides was identified for significant proteins in ventricle than atria in Doll *et al*., suggesting that protein degradation had a skewed effect on protein quantification across chambers. The bar graphs at the bottom show type of protein identification in the dataset by Doll *et al.* for significant proteins. Proteins deemed significant in both our study and theirs are to a larger extent based on MS/MS identifications **d-e.** Examples of proteins deemed significant in ventricle (e) or atria (f) from Doll *et al.* where declaration of significance are affected by the imputation strategy and match-between-runs: e. g. no MS/MS measurements in the cardiac chambers, significance caused by narrow distribution of imputed values (red), and/or the best evidence for a protein's existence (MS/MS identification, beige) identified in samples of the opposite cardiac region than where the protein was deemed significant **f.** Examples of proteins where isoforms are simultaneously identified as significant in both atria *and* ventricle by Doll *et al*. Only identifications in the atria were based on high-quality data. Significance in ventricle was based on matched values and driven by low-variance imputed values. Inclusion of isoform information in the quantitative analyses and unreviewed Trembl-based protein identifications causes significance of the low-confidence truncated TUBB3 sequence Q13509 (46 amino acids fragment of full-length TUBB3) in ventricle. **Abbreviations**: Protein

intensity: $\log_2$(LFQ intensity) in Doll *et al*. except lower panel c: normalized raw protein intensity. LA: left atrium, RA: right atrium, LV: left ventricle, RV: right ventricle, SepA: atrial septum, SepV: ventricular septum.

Supplementary Figure S4

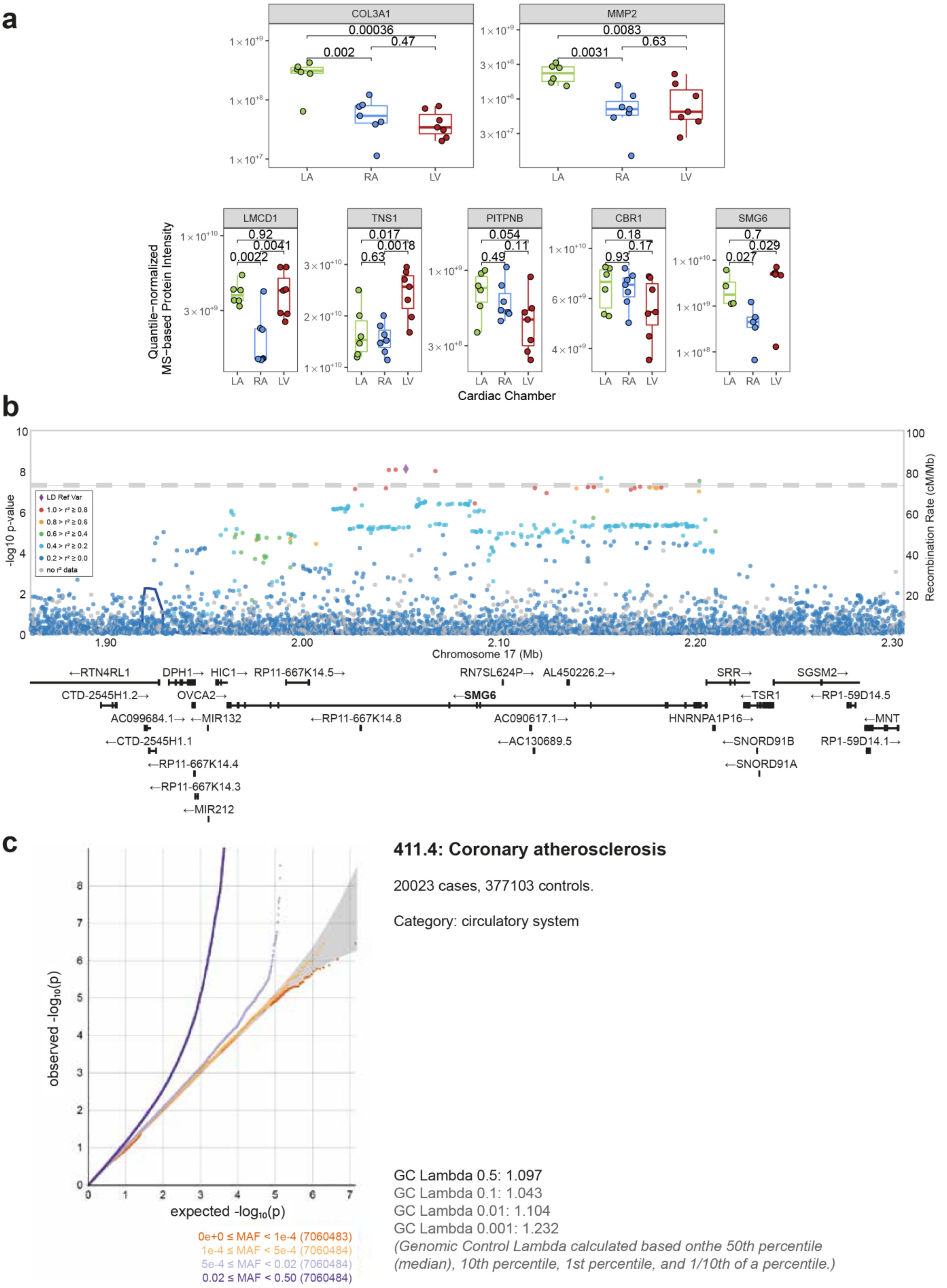

Supplementary Figure S4: Protein abundance of mitral valve disease associated genes indicates chamber-specific functionality. a. Protein intensity of COL3A1, MMP2 , LMCD1, TNS1, PITPNB, CBR1, and SMG6 across cardiac chambers. COL3A1 and MMP2, which are involved in mitral valve disease, show significantly higher expression in LA. The two proteins previously confirmed to be involved in MVD (LMCD1,TNS1) are more highly abundant in the LV. Proteins we suggest as likely causal genes in the respective locus are expressed at comparable levels across chambers (CBR1, PITPNB), or differentially expressed across all chambers (SMG6). Box plots depict group median (line), inter-quartile range (box, 25th-75th percentile, IQR), and 1.5x IQR (whiskers, for outlier detection). P-values: two-sided Student’s t-test p-values **b.** Linkage disequilibrium (LD) plot showing genome-wide significance of SNPs in SMG6 gene retrieved from PheWeb **c.** QQ Plot for coronary atherosclerosis retrieved from PheWeb shows clear signal for genetic association of the phenotype: Significant p-values are over-represented for disease associated SNPs with minor allele frequency (MAF) above 5E-4 (purple points) when compared to the expected distribution (on the diagonal). Analysis was performed on 20,023 cases and 377,103 controls. Genomic inflation factor ʎ at the minor allele frequency for 0.01 p-value percentile was 1.104.

Supplementary Figure S5

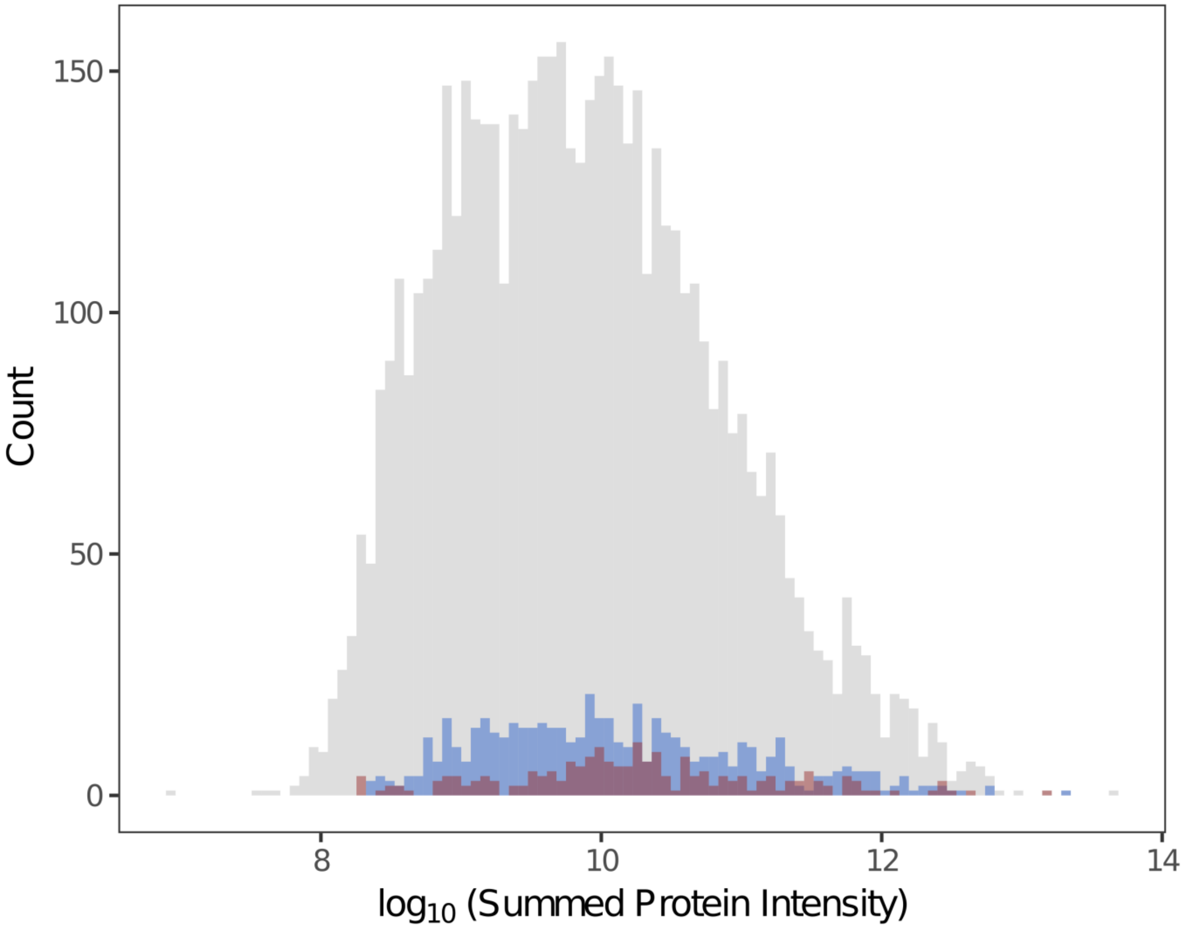

Supplementary Figure S5: Intensity distribution of significant proteins between atria and ventricles. Histogram of protein intensities of all proteins quantified in the volcano plot (grey), as well as proteins significant in atria (blue) and ventricle (red). Intensity distributions show that both high and low abundant proteins are deemed significant.

Supplementary Figure S6

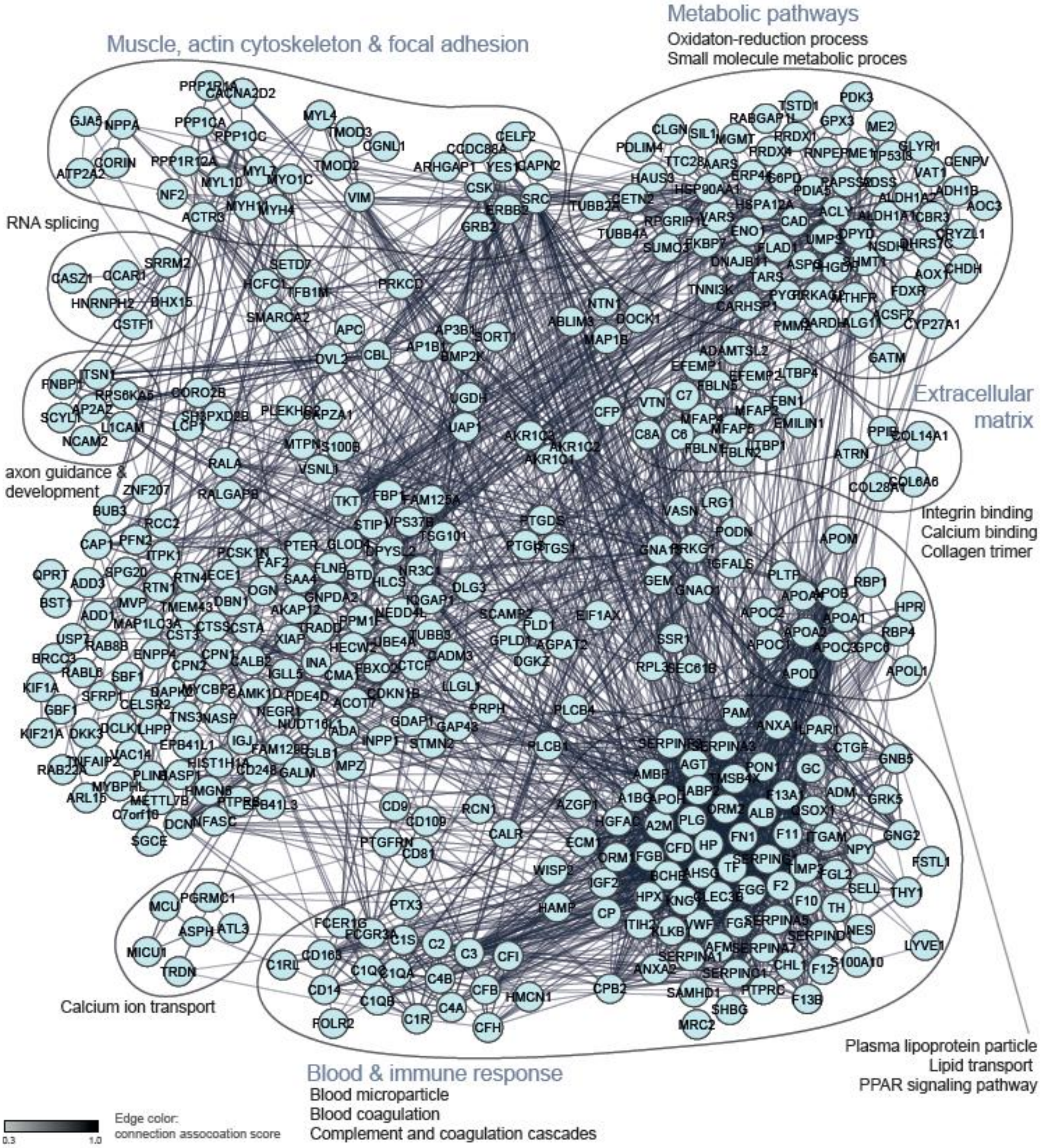

Supplementary Figure S6: Protein association network of proteins enriched in human atria reveal molecular components of tissue specialization including higher abundance of muscle, metabolic processes and plasma proteins. Network analysis of proteins significantly enriched in atria. A protein-protein interaction network was generated for all proteins with atria-specific expression. Proteins are represented by nodes, where the node size reflects measured protein abundance in the atria. The network was grouped by clustering

of proteins according to strength of the interaction score. For each cluster, gene ontology (GO) terms and KEGG pathway associations were retrieved and resulted in functional enrichments as summarized in the figure.

Supplementary Figure S7

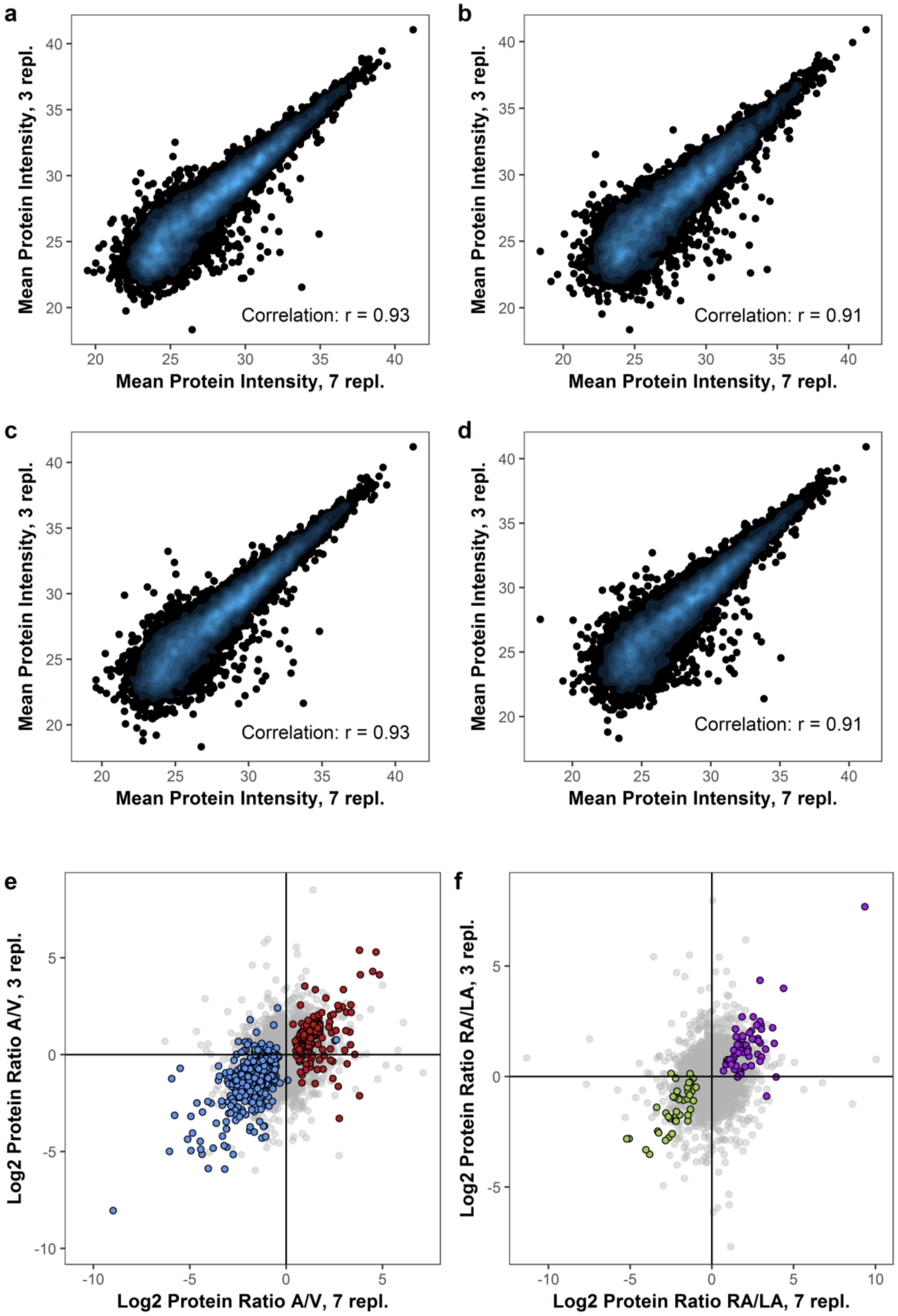

Supplementary Figure S7: Validation of proteomics findings with an independently acquired dataset. Additionally to the seven primary replicates, we independently repeated measurements with three further replicates to confirm findings based on proteomics data. **a-d.** Correlation of mean normalized protein intensity data between datasets for (a) both atria pooled, (b) left ventricle, (c) right atrium, and (d) left atrium. Color scale shows point density (light blue = highest point density, black = lowest point density). **e-f.** Comparison of fold-change protein expression between datasets for (e) atrial versus ventricular protein expression, and (f) right versus left atrial protein expression. Proteins deemed significant in the primary dataset are highlighted in red (ventricle), blue (atria), purple (right atrium) and green (left atrium). In all plots, values from the primary dataset are plotted on the x-axis, and values from the validation dataset on the y-axis. A: atria; V: ventricle, RA: right atrium, LA: left atrium, repl.: replicate.

Supplementary Figure S8

a

Gene name	No. of variants in ExAC	No. of DCM variants in HGMD	Fraction exclusively in HGMD	Confidence score *	DCM Confidence bin	Protein expression ratio (LV/atria)	Log_{10} (Median Protein Intensity in LV)
LMNA	11	80	0.86	59.51	high	0.8	11.0
MYH7	20	71	0.72	36.63	high	1.7	12.0
TTN	15	55	0.73	29.09	high	1.0	11.4
TNNT2	4	20	0.80	12.80	high	1.0	11.4
MYBPC3	13	32	0.59	11.28	high	1.3	11.5
RBM20	3	16	0.81	10.56	high	1.4	8.4
TPM1	1	10	0.90	8.10	high	1.0	11.4
TNNI3	1	8	0.88	6.13	high	1.1	11.1
DMD	2	9	0.78	5.44	high	0.8	10.8
BAG3	6	15	0.60	5.40	high	0.7	9.7
DSP	2	8	0.75	4.50	high	2.6	10.8
SCN5A	3	8	0.63	3.13	high	0.8	8.3
MYPN	5	10	0.50	2.50	high	1.6	10.0
ANKRD1	2	5	0.60	1.80	high	0.8	9.9
RAF1	3	6	0.50	1.50	high	7.3	8.4
LDB3	8	12	0.33	1.33	high	1.0	11.2
DES	5	8	0.38	1.13	high	1.1	11.3
PLN	3	5	0.40	0.80	medium	2.0	9.5
NEXN	1	2	0.50	0.50	medium	1.8	10.1
LAMA2	1	2	0.50	0.50	medium	1.5	9.9
TXNRD2	1	2	0.50	0.50	medium	1.2	9.7
CRYAB	2	3	0.33	0.33	medium	0.9	11.0
MYH6	3	4	0.25	0.25	medium	1.0	12.3
VCL	3	4	0.25	0.25	medium	1.0	11.1
ACTN2	3	4	0.25	0.25	medium	1.4	10.7
NEBL	4	4	0	0	low	1.6	10.9
MURC	4	4	0	0	low	0.6	10.0
CSRP3	3	3	0	0	low	1.3	11.1
LAMA4	2	2	0	0	low	1.4	9.4
FLNC	1	1	0	0	low	2.2	11.3
SGCD	1	1	0	0	low	1.0	9.9
TMPO	1	1	0	0	low	0.7	8.9
SYNE1	1	1	0	0	low	0.9	8.8
FHOD3	1	1	0	0	low	1.2	8.7
VPS13A	1	1	0	0	low	0.5	8.5
FLT1	1	1	0	0	low	1.2	8.4
ABCC9	1	1	0	0	low	0.9	7.2

low — high

* Confidence score = (No. of HGMD DCM variants - No. of ExAC variants) * Fraction of variants only found in HGMD

b

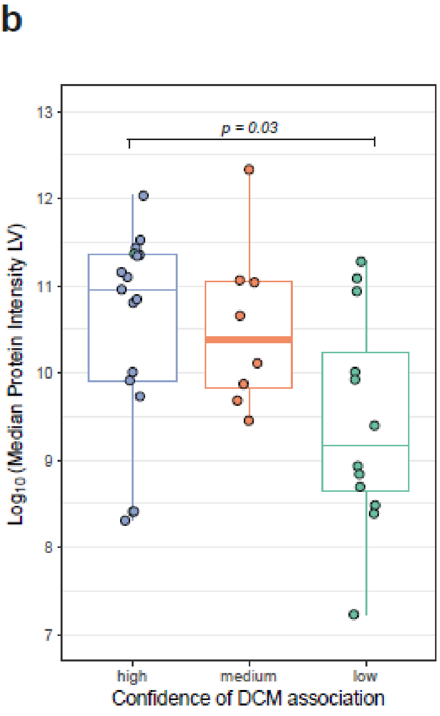

c

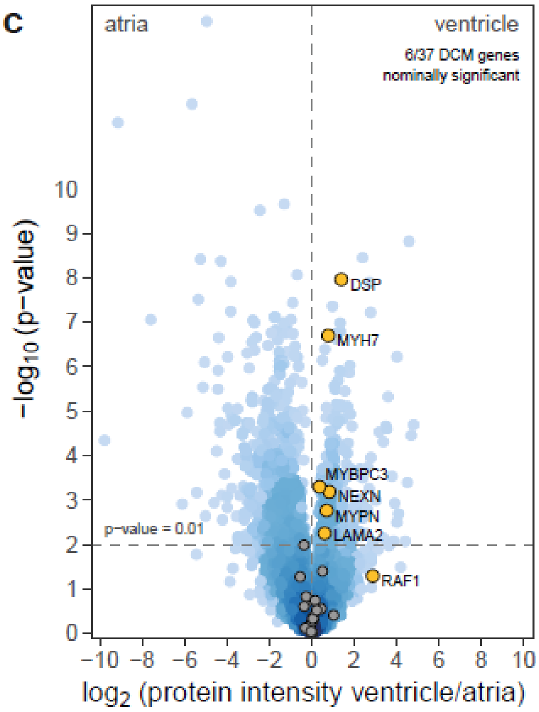

Supplementary Figure S8: Protein expression of genetic associations to Dilated Cardiomyopathy (DCM) clearly distinguishes "high" from "low" confidence genetic associations. a. Rare mutation-based DCM-associated genes as reviewed by Nouravesh *et al*. (2016). DCM associations were classified into confidence bins based on a confidence score calculated from the prevalence of de novo mutations in DCM patients and the corresponding prevalence in the general population. Protein abundance in human left ventricle (LV) was extracted from our data. **b.** Box plots show protein intensities from panel a for each level of confidence. Protein abundance of high confidence variants is significantly higher compared to low confidence variants (p: two-sided Student's t-test p-value). Line decpits group median, box depicts inter-quartile range (25th-75th percentile, IQR), whiskers depict 1.5x IQR (for outlier detection) **c.** Volcano plot from Fig. 4 showing differential proteins expression between atria and ventricle. DCM variants found in our data are highlighted. Six genetic DCM variants were higher expressed in human ventricle at nominal significance ($p<0.01$, two-sided Students t-test). *HGMD*: Human Gene Mutation Database. *ExAC*: Exome Aggregation Consortium database of aggregated exome sequencing data.

Supplementary Figure S9

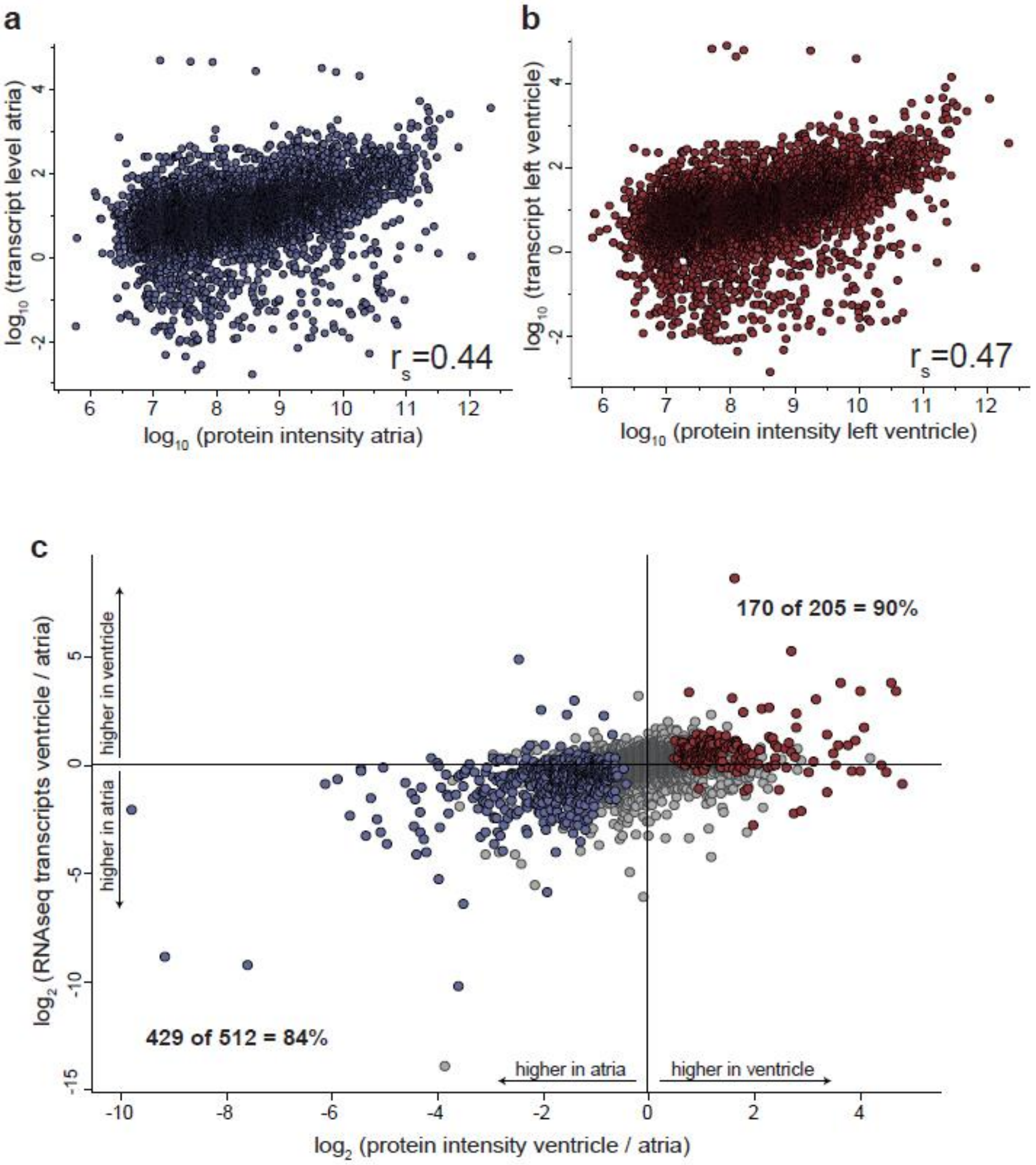

Supplementary Figure S9: Correlation between transcript expression levels (GTEx) and protein expression levels supports generalizability of protein expression to population level. a. Correlation plot of normalized protein intensities measured in human atria and transcript levels (transcripts per billion) in atria retrieved from GTEx **b.** Correlation plot of protein intensity in left ventricle and transcript levels in left ventricle retrieved from GTEx **c.** Transcript ratios between ventricle and atria (from GTEx) are plotted against protein intensity ratios between atria and ventricle from our data. The plot shows high consensus between protein expression ratios and transcript expression ratio (from GTEx), where the same

direction of regulation is observed for 84% of proteins significantly higher abundant in atria (blue) and 90% of proteins significantly higher abundant in ventricle (red). r_s: Spearman's rank correlation coefficient.

Supplementary Figure S10

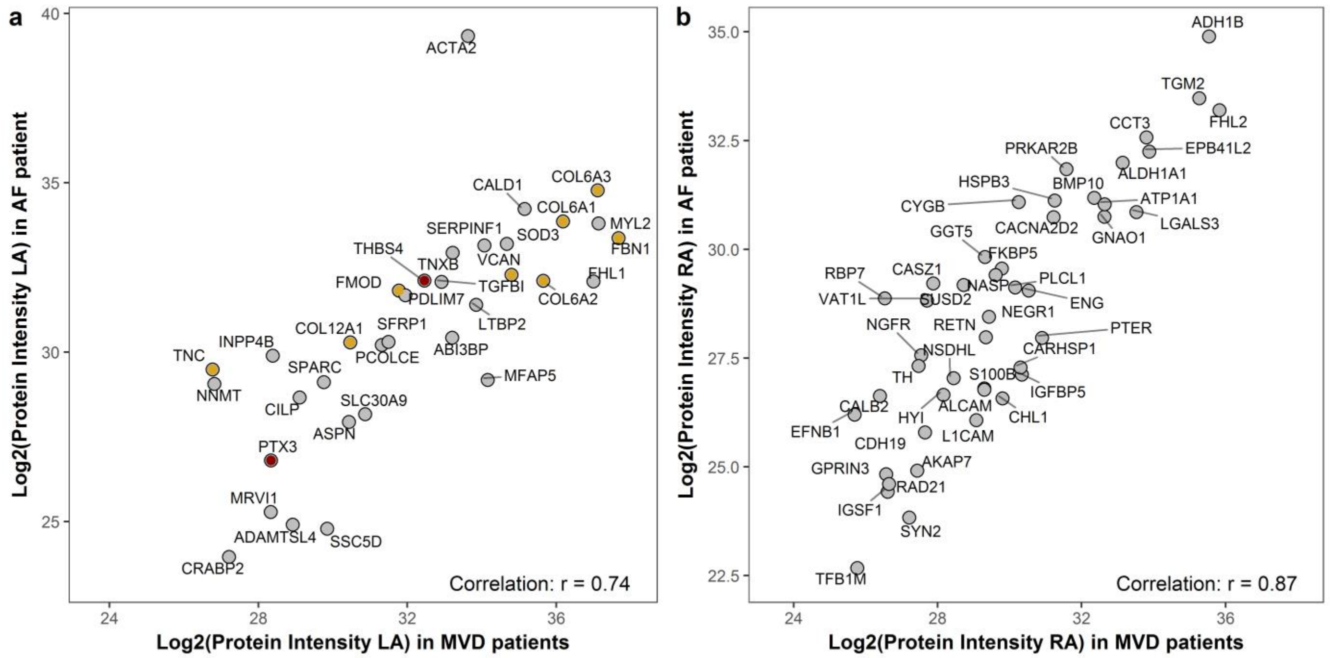

Supplementary Figure S10: Comparison of primary dataset with a heart affected by atrial fibrillation (AF). Mean normalized protein intensities for all proteins significantly differentially expressed in (a) left atrium (LA) and (b) right atrium (RA) from this study are plotted against protein intensity measurements independently obtained from the heart of an AF patient. Proteins discussed in the main manuscript text are highlighted in red (AF related) and yellow (fibrosis related).

Supplementary Tables

Supplementary Table S1: Patient info for the seven individuals included in the study. Abbreviations: *NYHA: New York Heart Association functional classification BMI: Body Mass Index, CAD: Coronary Artery Disease, PVD: Peripheral Vascular Disease, ECG: Electrocardiography, SR: Sinus Rhythm, ECG PR: PR duration, ECG QRS: QRS interval duration, ECG QTc: QTc interval duration, EKKO LVEF: Left Ventricular Ejection Fraction (%), alcohol in units per week, n. a.: not available*

Sample	Sex	Age at inclusion	NYHA	BMI	LA dilation	CAD	Hyper-tension	Diabetes	PVD	Stroke	ECG rhythm
1	M	60	2	24.1	severe	no	no	no	no	no	SR
2	M	50	2	26.6	severe	no	no	no	no	no	SR
3	M	44	2	24.8	moderate	no	no	no	no	no	SR
4	M	54	2	20.4	severe	no	no	no	no	no	SR
5	M	49	2	20.8	n. a.	no	no	no	no	yes	SR
6	M	43	2	24.7	severe	no	no	no	no	no	SR
7	M	50	1	24.0	severe	no	no	no	no	no	SR, 1st degree AV-block

Supplementary Table S1, continued:

Sample	ECG PR	ECG QRS	ECG QTc	EKKO LVEF	Medication	Smoking	Alcohol
1	176 ms	98 ms	428 ms	60	Lansoprazol, Ritalin	never	2
2	126 ms	98 ms	433 ms	60	Olanzapin, truxal, pinex, oxycodonhydrochlorid, morfin	yes, 50 pck yrs	0, earlier absuse
3	144 ms	88 ms	426 ms	60	none	never	5
4	160 ms	96 ms	398 ms	60	none	never	21
5	186 ms	118 ms	426 ms	60	Atorvastatin, clopidogrel (begge pause 5 dage inden OP)	yes, 30 pck yrs	7
6	134 ms	80 ms	383 ms	60	none	previously, 7 pck yrs	12
7	232 ms	96 ms	392 ms	60	none	never	2

Legends for Supplementary Tables S2-S4

Supplementary Table S2: Proteins identified in cardiac biopsies of left ventricle, and left and right atrium collected *in vivo* from seven individuals during surgery. A total of 7314 protein groups were identified across chambers of which 6999 were identified with at least 2 peptides.

Supplementary Table S3: Significantly different proteins between human atria and left ventricle as determined by volcano plot analysis.

Supplementary Table S4: Significantly different proteins between human right and left atrium as determined by volcano plot analysis.

References

1. Bekker-Jensen DB, Kelstrup CD, Batth TS, Larsen SC, Haldrup C, Bramsen JB, Sørensen KD, Høyer S, Ørntoft TF, Andersen CL, Nielsen ML and Olsen JV. An Optimized Shotgun Strategy for the Rapid Generation of Comprehensive Human Proteomes. *Cell Systems*. 2017;4:587-599.e4.

2. Tyanova S, Temu T and Cox J. The MaxQuant computational platform for mass spectrometry-based shotgun proteomics. *Nature Protocols*. 2016;11:2301-2319.

3. Tyanova S, Temu T, Sinitcyn P, Carlson A, Hein MY, Geiger T, Mann M and Cox J. The Perseus computational platform for comprehensive analysis of (prote)omics data. *Nature Methods*. 2016;13:731-740.

4. Bolstad BM, Irizarry RA, Astrand M and Speed TP. A comparison of normalization methods for high density oligonucleotide array data based on variance and bias. *Bioinformatics*. 2003;19:185-93.

5. Shannon P, Markiel A, Ozier O, Baliga NS, Wang JT, Ramage D, Amin N, Schwikowski B and Ideker T. Cytoscape: a software environment for integrated models of biomolecular interaction networks. *Genome research*. 2003;13:2498-504.

6. Morris JH, Apeltsin L, Newman AM, Baumbach J, Wittkop T, Su G, Bader GD and Ferrin TE. clusterMaker: a multi-algorithm clustering plugin for Cytoscape. *BMC Bioinformatics*. 2011;12:436.

7. Szklarczyk D, Morris JH, Cook H, Kuhn M, Wyder S, Simonovic M, Santos A, Doncheva NT, Roth A, Bork P, Jensen LJ and von Mering C. The STRING database in 2017: quality-controlled protein–protein association networks, made broadly accessible. *Nucleic Acids Research*. 2017;45:D362-D368.

8. Nouhravesh N, Ahlberg G, Ghouse J, Andreasen C, Svendsen JH, Haunso S, Bundgaard H, Weeke PE and Olesen MS. Analyses of more than 60,000 exomes questions the role of numerous genes previously associated with dilated cardiomyopathy. *Mol Genet Genomic Med*. 2016;4:617-623.

9. Stenson PD, Mort M, Ball EV, Evans K, Hayden M, Heywood S, Hussain M, Phillips AD and Cooper DN. The Human Gene Mutation Database: towards a comprehensive repository of inherited mutation data for medical research, genetic diagnosis and next-generation sequencing studies. *Human Genetics*. 2017;136:665-677.

10. Lek M, Karczewski KJ, Minikel EV, Samocha KE, Banks E, Fennell T, O'Donnell-Luria AH, Ware JS, Hill AJ, Cummings BB, Tukiainen T, Birnbaum DP, Kosmicki JA, Duncan LE, Estrada K, Zhao F, Zou J, Pierce-Hoffman E, Berghout J, Cooper DN, Deflaux N, DePristo M, Do R, Flannick J, Fromer M, Gauthier L, Goldstein J, Gupta N, Howrigan D, Kiezun A, Kurki MI, Moonshine AL, Natarajan P, Orozco L, Peloso GM, Poplin R, Rivas MA, Ruano-Rubio V, Rose SA, Ruderfer DM, Shakir K, Stenson PD, Stevens C, Thomas BP, Tiao G, Tusie-Luna MT, Weisburd B, Won H-H, Yu D, Altshuler DM, Ardissino D, Boehnke M, Danesh J, Donnelly S, Elosua R, Florez JC, Gabriel SB, Getz G, Glatt SJ, Hultman CM, Kathiresan S, Laakso M, McCarroll S, McCarthy MI, McGovern D, McPherson R, Neale BM, Palotie A, Purcell SM, Saleheen D, Scharf JM, Sklar P, Sullivan PF, Tuomilehto J, Tsuang MT, Watkins HC, Wilson JG, Daly MJ, MacArthur DG and Exome Aggregation C. Analysis of protein-coding genetic variation in 60,706 humans. *Nature*. 2016;536:285.

11. Vizcaíno JA, Csordas A, del-Toro N, Dianes JA, Griss J, Lavidas I, Mayer G, Perez-Riverol Y, Reisinger F, Ternent T, Xu Q-W, Wang R and Hermjakob H. 2016 update of the PRIDE database and its related tools. *Nucleic Acids Research*. 2016;44:D447-D456.

12. Doll S, Dreßen M, Geyer PE, Itzhak DN, Braun C, Doppler SA, Meier F, Deutsch M-A, Lahm H, Lange R, Krane M and Mann M. Region and cell-type resolved quantitative proteomic map of the human heart. *Nature Communications*. 2017;8.
